# Conversion of di­aryl­chalcones into 4,5-di­hydro­pyrazole-1-carbo­thio­amides: mol­ecular and supra­molecular structures of two precursors and three products

**DOI:** 10.1107/S2056989020001735

**Published:** 2020-02-14

**Authors:** Mohammed A. E. Shaibah, Hemmige S. Yathirajan, Asma, Nagaraja Manju, Balakrishna Kalluraya, Ravindranath S. Rathore, Christopher Glidewell

**Affiliations:** a Department of Studies in Chemistry, University of Mysore, Manasagangotri, Mysuru-570 006, India; b Department of Studies in Chemistry, Mangalore University, Mangalagangotri, Mangalore-574 199, India; c Department of Bioinformatics, School of Earth, Biological and Environmental Sciences, Central University of South Bihar, Gaya-824236, India; d School of Chemistry, University of St Andrews, St Andrews, Fife KY16 9ST, UK

**Keywords:** synthesis, cyclo­condensation, chalcones, heterocyclic compounds, reduced pyrazoles, crystal structures, mol­ecular conformation, hydrogen bonding, supra­molecular assembly

## Abstract

Chalcones of type 4-*X*C_6_H_4_C(O)CH=CHC_6_H_4_(OCH_2_CCH)-4, where *X* = Cl, Br or MeO, have been converted to the corresponding 4,5-di­hydro­pyrazole-1-carbo­thio­amides using a cyclo­condensation reaction with thio­semicarbazide. The chalcones 1-(4-chloro­phen­yl)-3-[4-(prop-2-yn­yloxy)phen­yl]prop-2-en-1-one, C_18_H_13_ClO_2_, (I), and 1-(4-bromo­phen­yl)-3-[4-(prop-2-yn­yloxy)phen­yl]prop-2-en-1-one, C_18_H_13_BrO_2_, (II), are isomorphous, and their mol­ecules are linked into sheets by two independent C—H⋯π(arene) inter­actions, both involving the same aryl ring with one C—H donor approaching each face. In each of the products (*RS*)-3-(4-chloro­phen­yl)-5-[4-(prop-2-yn­yloxy)phen­yl]-4,5-di­hydro­pyrazole-1-carbo­thio­amide, C_19_H_16_ClN_3_OS, (IV), (*RS*)-3-(4-bromo­phen­yl)-5-[4-(prop-2-yn­yloxy)phen­yl]-4,5-di­hydro­pyrazole-1-carbo­thio­amide, C_19_H_16_BrN_3_OS, (V), and (*RS*)-3-(4-meth­oxy­phen­yl)-5-[4-(prop-2-yn­yloxy)phen­yl]-4,5-di­hydro­pyrazole-1-carbo­thio­amide, C_20_H_19_N_3_O_2_S, (VI), the reduced pyrazole ring adopts an envelope conformation with the C atom bearing the 4-prop-2-yn­yloxy)phenyl substituent, which occupies the axial site, displaced from the plane of the four ring atoms. Compounds (IV) and (V) are isomorphous and their mol­ecules are linked into chains of edge-fused rings by a combination of N—H⋯S and C—H⋯S hydrogen bonds. The mol­ecules of (VI) are linked into sheets by a combination of N—H⋯S, N—H⋯N and C—H⋯π(arene) hydrogen bonds. Comparisons are made with the structures of some related compounds.

## Abstract 2

1,3-Disubstituted chalcones have been converted into 3,5-disubstituted 4,5-di­hydro­pyrazole-1-carbo­thio­amides by reaction with thio­semicarbazide. Two isomorphous chalcone precursors form hydrogen-bonded sheets, while in two isomorphous reduced pyrazole products, hydrogen-bonded chains of rings are formed: in a third product, the mol­ecules are linked into complex sheets.

## Chemical context   

Pyrazole derivatives exhibit a wide range of pharmacological activities, including analgesic (Badawey & El-Ashmawey, 1998 ▸), anti­bacterial (Zhang *et al.*, 2017 ▸), anti­cancer (Koca *et al.*, 2013 ▸) and anti-inflammatory (Vijesh *et al.*, 2013 ▸) activity, and recent work on both the synthesis of pyrazole derivatives and their pharmacological activities has been reviewed recently (Karrouchi *et al.*, 2018 ▸). With this background in mind, we have now employed three chalcones, compounds (I)–(III) as precursors for the synthesis of the corresponding 4,5-di­hydro­pyrazole-1-carbo­thio­amides, compounds (IV)–(VI), and we report here the mol­ecular and supra­molecular structures of two of the chalcone precursors, compounds (I) and (II), and of the three reduced pyrazole products (IV)–(VI): unfortunately, we have been unable to obtain satisfactory crystals of the chalcone (III). The chalcones were prepared (Fig. 6 ▸) by base-promoted condensation (Yuan *et al.*, 2009 ▸; Yu *et al.*, 2016 ▸; Yadav *et al.*, 2017 ▸) of the appropriately substituted aceto­phenones with 4-(prop-2-yn­yloxy)benz­aldehye (Hans *et al.*, 2010 ▸). Subsequent base-promoted cyclo­addition of the chalcones (I)–(III) with thio­semicarbazide yielded the products (IV)–(VI).
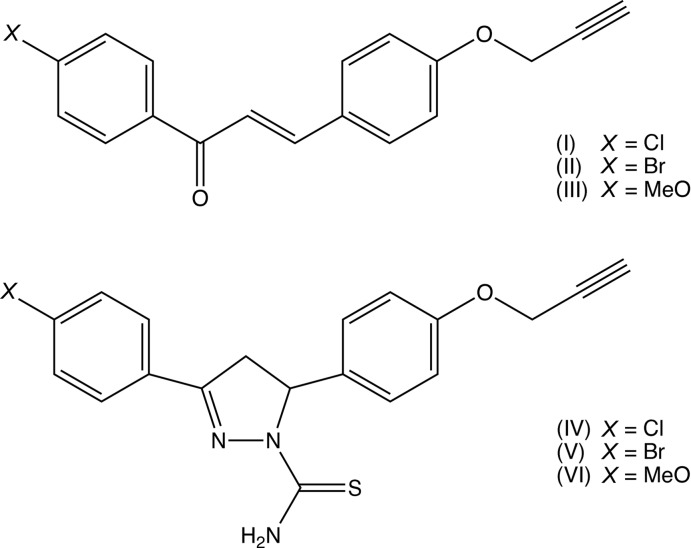


## Structural commentary   

**Figure 1:**
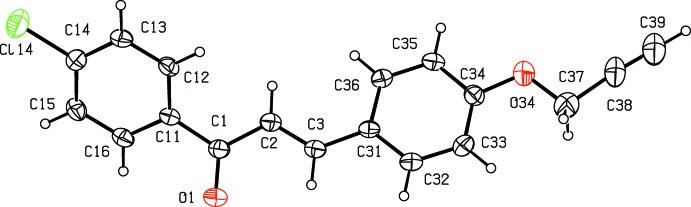
The mol­ecular structure of compound (I) showing the atom-labelling scheme. Displacement ellipsoids are drawn at the 30% probability level.

Compounds (I) and (II) are isomorphous in space group *P*2_1_/*c* (Fig. 1 ▸ & 2). In each of these two compounds, the non-H atoms, apart from those of the ring (C11–C16) are almost coplanar: the r.m.s. deviations from the mean planes through the atoms C1 to C39 (Figs. 1 ▸ and 2 ▸) are 0.0455 Å in (I) and 0.0617 Å in (II), with the maximum deviation from this plane exhibited in each case by atom C1, 0.087 (2) Å in (I) and 0.092 (3) Å in (II). On the other hand, the ring (C11–C16) is twisted out of this plane, making a dihedral angle with it of 44.6 (6)° in (I) and 44.47 (8)° in (II).

**Figure 3:**
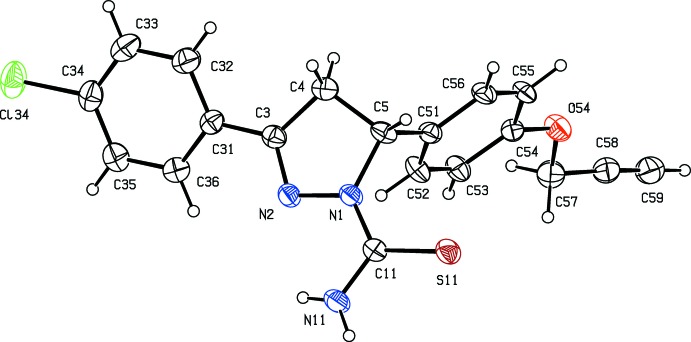
The mol­ecular structure of compound (IV) showing the atom-labelling scheme. Displacement ellipsoids are drawn at the 30% probability level.

**Figure 6:**
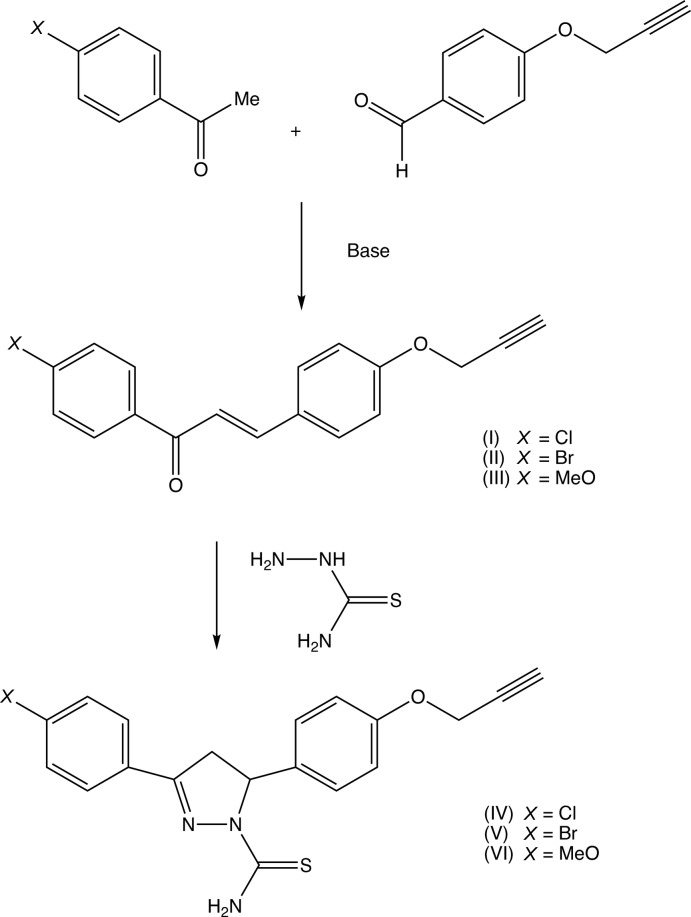
The synthetic route to compounds (I)–(VI).

Compounds (IV) and (V) are likewise isomorphous, this time in space group *P*2_1_/*n* (Figs. 3 ▸ and 4 ▸). In each of compounds (IV)–(VI), there is a stereogenic centre at atom C5 (Figs. 3 ▸–5 ▸ ▸) and, in each case, the reference mol­ecule was selected to be the one having the *R* configuration at this centre: the centrosymmetric space groups confirm that compounds (IV)–(VI) have all crystallized as racemic mixtures. The reduced pyrazole rings all adopt envelope conformations, folded across the line N1⋯C4: the ring-puckering parameters, calculated for the atom sequence (N1,N2,C3,C4,C5) are *Q*_2_ = 0.204 (3), 0.285 (4) and 0.217 (3) Å, and φ_2_ = 15.9 (10), 316.5 (12) and 319.4 (7)°, for (IV)–(VI), respectively. The displacements of the atom C5 from the plane of the other four atoms in the reduced pyrazole ring are 0.330 (5), 0.332 (6) and 0.351 (4) Å in compounds (IV)–(VI), respectively, and, in each case, the aryl substituent at atom C5 occupies the axial site. In compound (VI), the meth­oxy C atom is displaced from the plane of the adjacent aryl ring by only 0.215 (6) Å: associated with this near planarity, the two exocyclic O—C—C angles at atom C34 differ by almost 10°, as is frequently observed in near-planar alk­oxy­arene systems (Seip & Seip, 1973 ▸; Ferguson *et al.*, 1996 ▸).

## Supra­molecular features   

**Figure 7:**
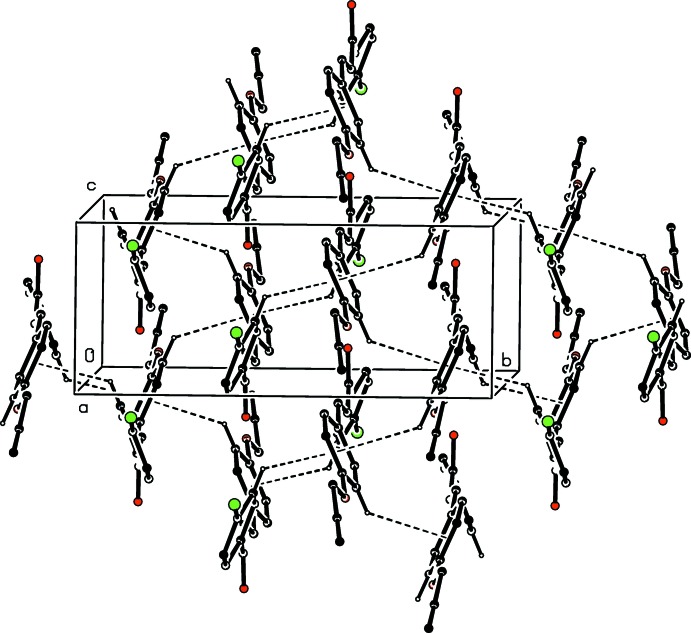
Part of the crystal structure of compound (I), showing the formation of a hydrogen-bonded sheet running parallel to (100). Hydrogen bonds are shown as dashed lines and, for the sake of clarity, the H atoms not involved in the motifs shown have been omitted.

Despite the presence of a carbonyl group in compounds (I) and (II), their structures do not contain any C—H⋯O hydrogen bonds (Table 1 ▸): there are no inter­molecular C⋯H contact distances less than 2.8 Å, well beyond the sum of the van der Waals radii, 2.68 Å (Rowland & Taylor, 1996 ▸). The structures do, however, contain two C—H⋯π(arene) hydrogen bonds, both involving the same ring (C31–C36) as the acceptor, with one C—H donor on each face of the ring and with H13^i^⋯*Cg*1⋯H35^ii^ angles of 158° in (I) and 157° in (II), where *Cg*1 represents the centroid of the (C31–C36) ring [symmetry codes: (i) 1 − *x*, 1 − *y*, 1 − *z*; (ii) *x*, 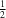 − *y*, 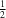 + *z*]. The combination of these two C—H⋯π hydrogen bonds links the mol­ecules into a sheet lying parallel to (100) and occupying the whole domain 0 < *x* < 1.0 (Fig. 7 ▸).

**Figure 8:**
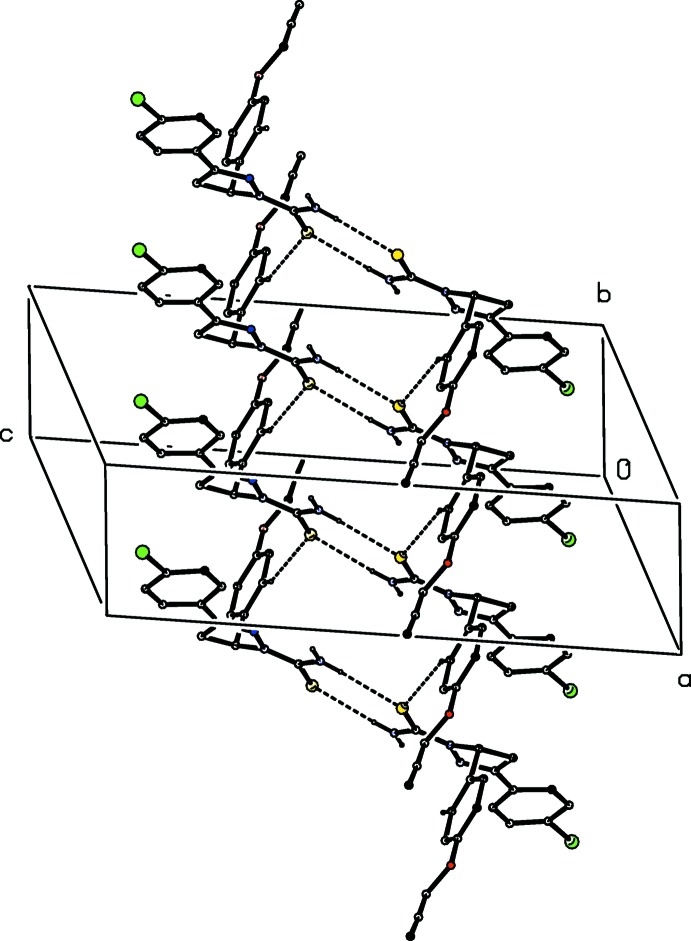
Part of the crystal structure of compound (IV), showing the formation of a hydrogen-bonded chain of rings lying parallel to [010]. Hydrogen bonds are shown as dashed lines and, for the sake of clarity, the H atoms not involved in the motifs shown have been omitted.

In each of the reduced pyrazole products (IV)–(VI) there is an intra­molecular N—H⋯N hydrogen bond (Table 1 ▸). In the isomorphous pair (IV) and (V), the mol­ecules are linked by a combination of N—H⋯S and C—H⋯S hydrogen bonds (Allen *et al.*, 1997 ▸) to form a ribbon in the form of a chain of centrosymmetric, edge-fused rings running parallel to the [010] direction, in which 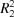
(8) (Etter, 1990 ▸; Etter *et al.*, 1990 ▸; Bernstein *et al.*, 1995 ▸) rings centred at (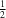, *n*, 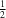) alternate with 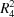
(18) rings centred at (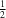, *n* + 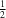, 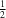), where *n* represents an integer in each case (Fig. 8 ▸). There is also a short N—H⋯Br contact in the structure of compound (V), but it has been shown from database analyses (Brammer *et al.*, 2001 ▸; Thallypally & Nangia, 2001 ▸) that halogen atoms bonded to C atoms are extremely poor acceptors of hydrogen bonds, so that this contact should not be regarded as structurally significant.

**Figure 9:**
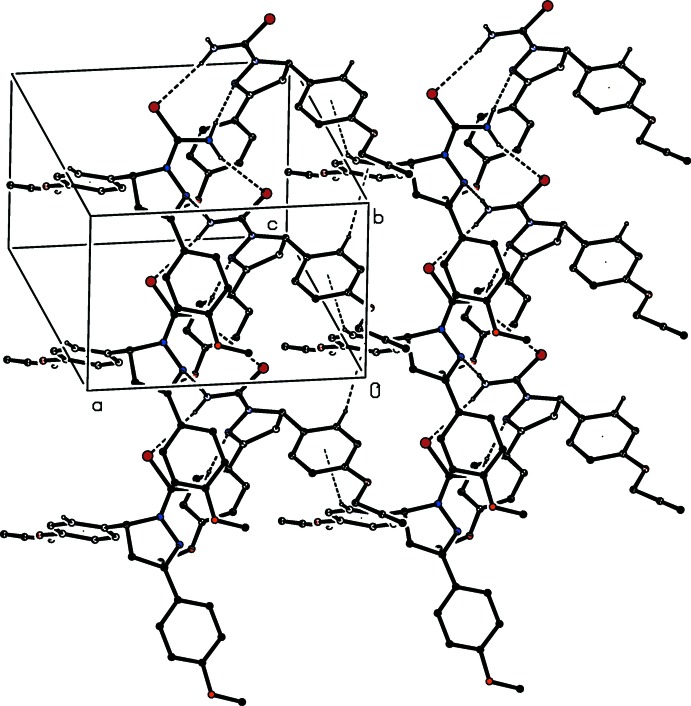
Part of the crystal structure of compound (VI), showing the formation of a hydrogen-bonded sheet running parallel to (001). Hydrogen bonds are shown as dashed lines and, for the sake of clarity, the H atoms not involved in the motifs shown have been omitted.

The mol­ecules of compound (VI) are linked by a combination of N—H⋯S, N—H⋯N and C—H⋯π(arene) hydrogen bonds to form a complex sheet lying parallel to (001) in the domain 0 < *z* < 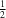 (Fig. 9 ▸): a second sheet, related to the first by inversion lies in the domain (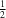 < *z* < 1.0). The only direction-specific inter­molecular contact between adjacent sheets is of the C—H⋯O type; however, this involves a C—H bond in a methyl group, which is probably undergoing fast rotation about the adjacent C—O bond (Riddell & Rogerson, 1996 ▸, 1997 ▸) and, in addition, it has a very small *D*—H⋯*A* angle, indicating a very small inter­action energy (Wood *et al.*, 2009 ▸). On both these grounds, this contact can be regarded as having negligible structural significance, so that the supra­molecular assembly in (VI) is two-dimensional.

## Database survey   

It is of inter­est to briefly compare the structures of the reduced pyrazole derivatives (IV)–(VI) reported here with those of some related compounds. Although there are no records of any 4,5-di­hydro­pyrazole-1-carbo­thio­amides recorded in the Cambridge Structural Database (CSD version 5.40, update of December 2019; Groom *et al.*, 2016 ▸), there are several examples of 4,5-di­hydro­pyrazole-1-carboxamides which contain a CONH_2_ substituent, as opposed to the CSNH_2_ substituent in compounds (IV)–(VI). Both 3-ethyl-5-hy­droxy-5- (tri­fluoro­meth­yl)-4,5-di­hydro­pyrazole-1-carboxamide (VII) (CSD refcode COJQUO; Sauzem *et al.*, 2008 ▸) and 5-hy­droxy-4-methyl-5-(tri­fluoro­meth­yl)-4,5-di­hydro­pyrazole-1-carboxamide (VIII) (COJRAV; Sauzem *et al.*, 2008 ▸) contain intra­molecular N—H⋯N hydrogen bonds analogous to those observed in compounds (IV)–(VI). In (VII), inversion-related pairs of mol­ecules are linked by paired N—H⋯O hydrogen bonds to form cyclic dimers characterized by an 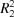
(8) motif, while in (VIII) a combination of O—H⋯O, N—H⋯O and N—H⋯N hydrogen bonds links the mol­ecules into complex sheets. In the enanti­opure disubstituted carboxamide (4*S*)-*N*-[4-(di­fluoro­meth­oxy)phen­yl]-4-(4-fluoro­phen­yl)-*N*-[(1*S*,4*R*)-4,7,7-trimethyl-3-oxo-2-oxabi­cyclo­(2.2.1)hept-1-ylcarbon­yl]-3-[4-(2,2,2-tri­fluoro­eth­oxy)phen­yl]-4,5-di­hydro­pyrazole-1-carboxamide (IX) (SOTBAE; Bosum-Dybus & Neh, 1991 ▸), the only inter­molecular hydrogen bonds are of the C—H⋯O type, and these link the mol­ecules into chains. We also note the structures of the simpler 4,5-di­hydro­pyrazoles 3-(2-naphth­yl)-5-hy­droxy-5-(tri­fluoro­meth­yl)-4,5-di­hydro­pyrazole (X) (MAFVUL; Yang & Raptis, 2003 ▸) and 3-(2,2-di­cyano­ethen­yl)-1-phenyl-4,5-di­hydro-1*H*-pyrazole (XI) (XEHMOM; Cole *et al.*, 2000 ▸), which is a non-linear-optical material crystallizing in space group *Cc*, and which has been the subject of a variable-temperature study employing both X-ray and neutron diffraction. Finally, we note that structures have been reported for a number of reduced 3,4′-bi­pyrazoles (Cuartas *et al.*, 2017 ▸; Kiran Kumar *et al.*, 2019 ▸).

## Synthesis and crystallization   

Samples of the chalcones (I)–(III) were prepared using the published methods (Hans *et al.*, 2010 ▸; Yuan *et al.*, 2009 ▸; Yu *et al.*, 2016 ▸; Yadav *et al.*, 2017 ▸): crystals of compounds (I) and (II), which were suitable for single-crystal X-ray diffraction, were grown by slow evaporation, at ambient temperature and in the presence of air from a solution in methanol. Despite repeated attempts, no suitable crystals of (III) could be obtained.

For the synthesis of compounds (IV)–(VI), a solution of potassium hydroxide (0.2 g) in ethanol (20 ml) was added to a mixture of thio­semicarbazide (140 mg, 1.5 mol) and the corresponding chalcone (I)–(III) (1 mmol). These mixtures were then heated under reflux for 5 h, when thin-layer chromatography indicated that the reactions were complete. The mixtures were then allowed to cool to ambient temperature, and the resulting solid products were collected by filtration, washed with water, dried in air and crystallized from a mixture of ethanol and *N*,*N*-di­methyl­formamide (9:1, *v*/*v*) to give the products (IV)–(VI).

Compound (IV). Yield 81%, m. p. 421 K. Analysis found C 61.7, H 4.4, N 11.4%; C_19_H_16_ClN_3_OS requires C 61.7, H 4.4, N 11.4%. IR (KBr, cm^−1^) 3440 (NH), 2123 (C≡C). NMR (DMSO-*d*_6_) δ(^1^H) 3.09 (*dd*, 1H *J* = 18.0, 3.3 Hz) and 3.84 (*dd*, *J* = 18.0, 11.5 Hz) (pyrazole CH_2_), 3.32 (*t*, 1H, *J* = 2.4 Hz, ≡C—H), 4.56 (*d*, 2H, *J* = 2.4 Hz OCH_2_), 5.73 (*dd*, 1H, *J* = 11.5, 3.3 Hz, pyrazole CH), 6.65 (*d*, 2H, *J* = 8.6 Hz) and 7.10 (*d*, 2H, *J* = 8.6 Hz) (C_6_H_4_O), 7.2 (*m*, 4H,C_6_H_4_Cl).

Compound (V). Yield 71%, m. p. 455–457 K. Analysis found C 55.2, H 3.9, N 10.1%; C_19_H_16_BrN_3_OS requires C 55.1, H 3.9, N 10.1%. IR (KBr, cm^−1^) 3414 (NH), 2126 (C≡C). NMR (DMSO-*d*_6_) δ(^1^H) 3.09 (*dd*, 1H *J* = 18.0, 3.4 Hz) and 3.80 (*dd*, *J* = 18.0, 11.5 Hz) (pyrazole CH_2_), 3.32 (*t*, 1H, *J* = 2.2 Hz, ≡C—H), 4.70 (*d*, 2H, *J* = 2.2 Hz OCH_2_), 5.89 (*dd*, 1H, *J* = 11.5, 3.4 Hz, pyrazole CH), 6.88 (*d*, 2H, *J* = 8.6 Hz) and 7.07 (*d*, 2H, *J* = 8.6 Hz) (C_6_H_4_O), 7.58 (*d*, 2H, *J* = 8.5 Hz) and 8.56 (*d*, 2H, *J* = 8.5 Hz) (C_6_H_4_Br).

Compound (VI). Yield 79%, m. p. 422–423 K. Analysis found C 65.8, H 5.2, N 11.5%; C_20_H_19_N_3_O_2_S requires C 65.7, H 5.2, N 11.5%. IR (KBr, cm^−1^) 3339 (NH), 2120 (C≡C). NMR (DMSO-*d*_6_) δ(^1^H) 3.09 (*dd*, 1H *J* = 17.9, 3.2 Hz) and 3.71 (*dd*, *J* = 17.0, 11.5 Hz) (pyrazole CH_2_), 3.69 (*t*, 1H, *J* = 2.3 Hz, ≡C—H), 3.78 (*s*, 3H, OC_h_), 4.52 (*d*, 2H, *J* = 2.3 Hz OCH_2_), 5.76 (*dd*, 1H, *J* = 11.5, 3.2 Hz, pyrazole CH), 6.75 (*d*, 2H, *J* = 8.8 Hz) and 7.02 (*d*, 2H, *J* = 8.8 Hz) (C_6_H_4_OCH_2_), 7.13 (*d*, 2H, *J* = 8.1 Hz) and 7.63 (*d*, 2H, *J* = 8.1 Hz) (C_6_H_4_OCH_3_).

Crystals of compounds (IV)–(VI), which were suitable for single-crystal X-ray diffraction analysis, were selected directly from the analytical samples.

## Refinement   

Crystal data, data collection and structure refinement details are summarized in Table 2 ▸. All H atoms were located in difference maps, and then treated as riding atoms in geometrically idealized positions with C—H distances of 0.93 Å (alkenyl, alkynyl and aromatic), 0.96 Å (CH_3_), 0.97 Å (CH_2_) or 0.98 Å (aliphatic C—H), and with *U*_iso_(H) = *kU*_eq_(C), where *k* = 1.5 for the methyl group, which was permitted to rotate but not to tilt, and 1.2 for all other H atoms.

## Supplementary Material

Crystal structure: contains datablock(s) global, I, II, IV, V, VI. DOI: 10.1107/S2056989020001735/zl2770sup1.cif

Structure factors: contains datablock(s) I. DOI: 10.1107/S2056989020001735/zl2770Isup2.hkl

Structure factors: contains datablock(s) II. DOI: 10.1107/S2056989020001735/zl2770IIsup3.hkl

Structure factors: contains datablock(s) IV. DOI: 10.1107/S2056989020001735/zl2770IVsup4.hkl

Structure factors: contains datablock(s) V. DOI: 10.1107/S2056989020001735/zl2770Vsup5.hkl

Structure factors: contains datablock(s) VI. DOI: 10.1107/S2056989020001735/zl2770VIsup6.hkl

Supporting information file. DOI: 10.1107/S2056989020001735/zl2770Isup7.cml

Supporting information file. DOI: 10.1107/S2056989020001735/zl2770IIsup8.cml

Supporting information file. DOI: 10.1107/S2056989020001735/zl2770IVsup9.cml

Supporting information file. DOI: 10.1107/S2056989020001735/zl2770Vsup10.cml

Supporting information file. DOI: 10.1107/S2056989020001735/zl2770VIsup11.cml

CCDC references: 1982489, 1982488, 1982487, 1982486, 1982485

Additional supporting information:  crystallographic information; 3D view; checkCIF report

## Figures and Tables

**Figure 2:**
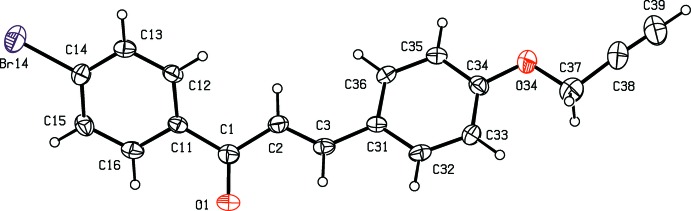
The mol­ecular structure of compound (II) showing the atom-labelling scheme. Displacement ellipsoids are drawn at the 30% probability level.

**Figure 4:**
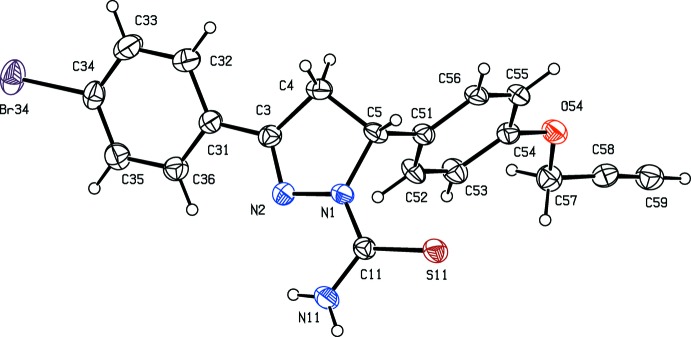
The mol­ecular structure of compound (V) showing the atom-labelling scheme. Displacement ellipsoids are drawn at the 30% probability level.

**Figure 5:**
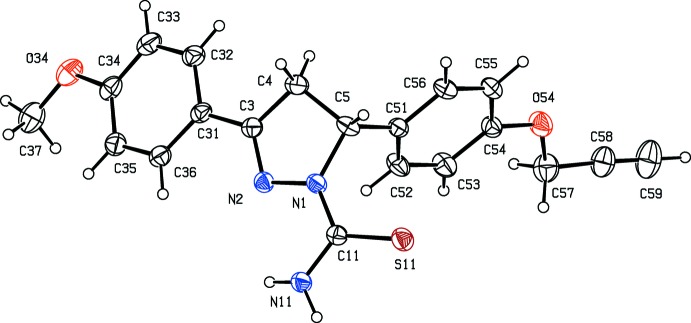
The mol­ecular structure of compound (VI) showing the atom-labelling scheme. Displacement ellipsoids are drawn at the 30% probability level.

**Table 1:** Hydrogen bonds and short intra- and inter mol­ecular contacts (Å, °) for compounds (I), (II) and (IV)–(VI) *Cg*1 and *Cg*2 represent the centroids of the rings (C31–C36) and (C51–C56), respectively

Compound	*D*—H⋯*A*	*D*—H	H⋯*A*	*D*⋯*A*	*D*—H⋯*A*
(I)	C13—H13⋯*Cg*1^i^	0.93	2.90	3.554 (3)	128
	C35—H35⋯*Cg*1^ii^	0.93	2.83	3.508 (3)	131
					
(II)	C13—H13⋯*Cg*1^i^	0.93	2.95	3.602 (4)	128
	C35—H35⋯*Cg*1^ii^	0.93	2.80	3.484 (3)	131
					
(IV)	N11—H11*A*⋯N2	0.80 (4)	2.23 (4)	2.614 (5)	110 (4)
	N11—H11*B*⋯S11^iii^	0.88 (4)	2.63 (4)	3.483 (4)	164 (4)
	C52—H52⋯S11^iv^	0.93	2.85	3.641 (4)	144
					
(V)	N11—H11*A*⋯N2	0.82 (5)	2.24 (6)	2.611 (5)	108 (5)
	N11—H11*A*⋯Br34^v^	0.82 (5)	2.89 (6)	3.632 (5)	152 (5)
	N11—H11*B*⋯S11^iii^	0.83 (6)	2.70 (6)	3.500 (5)	162 (6)
	C52—H52⋯S11^iv^	0.93	2.87	3.650 (4)	143
					
(VI)	N11—H11*A*⋯N2	0.88 (2)	2.32 (2)	2.637 (3)	101.2 (18)
	N11—H11*A*⋯S11^vi^	0.88 (2)	2.68 (2)	3.474 (2)	151 (2)
	N11—H11*B*⋯N2^vii^	0.89 (2)	2.17 (2)	3.049 (3)	175 (2)
	C37—H37*B*⋯O34^viii^	0.96	2.55	3.302 (4)	135
	C56—H56⋯*Cg*2^ix^	0.93	2.93	3.717 (3)	143

Symmetry codes: (i) 1 − *x*, 1 − *y*, 1 − *z*; (ii) *x*, 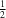 − *y*, 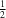 + *z*; (iii) 1 − *x*, 2 − *y*, 1 − *z*; (iv) *x*, −1 + *y*, *z*; (v) 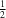 − *x*, 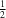 + *y*, 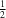 − *z*; (vi) 1 − *x*, −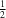 + *y*, 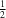 − *z*; (vii) 1 − *x*, 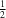 + *y*, 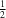 − *z*; (viii) 1 − *x*, −1 − *y*, 1 − *z*; (ix) −*x*, 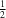 + *y*, 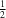 − *z*.

**Table 2:** Experimental details

	(I)	(II)	(IV)	(V)	(VI)
Crystal data					
Chemical formula	C_18_H_13_ClO_2_	C_18_H_13_BrO_2_	C_19_H_16_ClN_3_OS	C_19_H_16_BrN_3_OS	C_20_H_19_N_3_O_2_S
*M*_r_	296.73	341.18	369.86	414.31	365.44
Crystal system, space group	Monoclinic, *P*2_1_/*c*	Monoclinic, *P*2_1_/*c*	Monoclinic, *P*2_1_/*n*	Monoclinic, *P*2_1_/*n*	Monoclinic, *P*2_1_/*c*
Temperature (K)	296	296	298	296	296
*a*, *b*, *c* (Å)	17.990 (3), 14.2529 (16), 5.8661 (8)	18.286 (6), 14.277 (4), 5.8489 (17)	15.0182 (9), 6.0579 (3), 20.8286 (12)	15.1255 (13), 6.0426 (5), 21.026 (2)	11.7852 (15), 7.5345 (11), 20.599 (3)
β (°)	94.419 (4)	94.521 (7)	110.573 (2)	110.555 (3)	93.555 (4)
*V* (Å^3^)	1499.7 (3)	1522.2 (8)	1774.11 (17)	1799.4 (3)	1825.6 (4)
*Z*	4	4	4	4	4
Radiation type	Mo *K*α	Mo *K*α	Mo *K*α	Mo *K*α	Mo *K*α
μ (mm^−1^)	0.26	2.70	0.35	2.41	0.20
Crystal size (mm)	0.20 × 0.20 × 0.15	0.20 × 0.15 × 0.15	0.20 × 0.15 × 0.10	0.20 × 0.15 × 0.10	0.20 × 0.20 × 0.15

Data collection					
Diffractometer	Bruker APEXII	Bruker APEXII	Bruker APEXII	Bruker APEXII	Bruker APEXII
Absorption correction	Multi-scan (*SADABS*; Bruker, 2012 ▸)	Multi-scan (*SADABS*; Bruker, 2012 ▸)	Multi-scan (*SADABS*; Bruker, 2012 ▸)	Multi-scan (*SADABS*; Bruker, 2012 ▸)	Multi-scan (*SADABS*; Bruker, 2012 ▸)
*T*_min_, *T*_max_	0.895, 0.962	0.491, 0.667	0.870, 0.966	0.584, 0.786	0.908, 0.971
No. of measured, independent and observed [*I* > 2σ(*I*)] reflections	20193, 2912, 1777	23006, 2945, 1335	25833, 3326, 2571	18295, 3365, 2559	20467, 3822, 1864
*R*_int_	0.048	0.119	0.064	0.053	0.100
(sin θ/λ)_max_ (Å^−1^)	0.614	0.621	0.607	0.607	0.631

Refinement					
*R*[*F* ^2^ > 2σ(*F* ^2^)], *wR*(*F* ^2^), *S*	0.055, 0.132, 1.05	0.043, 0.089, 1.00	0.075, 0.133, 1.24	0.055, 0.113, 1.16	0.051, 0.118, 0.97
No. of reflections	2912	2945	3326	3365	3822
No. of parameters	190	190	232	232	242
H-atom treatment	H-atom parameters constrained	H-atom parameters constrained	H atoms treated by a mixture of independent and constrained refinement	H atoms treated by a mixture of independent and constrained refinement	H atoms treated by a mixture of independent and constrained refinement
Δρ_max_, Δρ_min_ (e Å^−3^)	0.32, −0.23	0.43, −0.50	0.19, −0.28	0.50, −0.46	0.21, −0.24

Computer programs: *APEX2*, *SAINT* and *XPREP* (Bruker, 2012 ▸), *SHELXT2014/5* (Sheldrick, 2015*a* ▸), *SHELXL2014* (Sheldrick, 2015*b* ▸) and *PLATON* (Spek, 2020 ▸).

## supplementary crystallographic information

### 1-(4-Chlorophenyl)-3-[4-(prop-2-ynyloxy)phenyl]prop-2-en-1-one (I) Crystal data

**Table:**

C_18_H_13_ClO_2_	*F*(000) = 616
*M**_r_* = 296.73	*D*_x_ = 1.314 Mg m^−^^3^
Monoclinic, *P*2_1_/*c*	Mo *K*α radiation, λ = 0.71073 Å
*a* = 17.990 (3) Å	Cell parameters from 2914 reflections
*b* = 14.2529 (16) Å	θ = 1.1–25.9°
*c* = 5.8661 (8) Å	µ = 0.26 mm^−^^1^
β = 94.419 (4)°	*T* = 296 K
*V* = 1499.7 (3) Å^3^	Block, orange
*Z* = 4	0.20 × 0.20 × 0.15 mm

### 1-(4-Chlorophenyl)-3-[4-(prop-2-ynyloxy)phenyl]prop-2-en-1-one (I) Data collection

**Table:**

Bruker APEXII diffractometer	2912 independent reflections
Radiation source: fine focussealed tube	1777 reflections with *I* > 2σ(*I*)
Graphite monochromator	*R*_int_ = 0.048
φ and ω scans	θ_max_ = 25.9°, θ_min_ = 2.3°
Absorption correction: multi-scan (SADABS; Bruker, 2012)	*h* = −21→22
*T*_min_ = 0.895, *T*_max_ = 0.962	*k* = −17→17
20193 measured reflections	*l* = −7→7

### 1-(4-Chlorophenyl)-3-[4-(prop-2-ynyloxy)phenyl]prop-2-en-1-one (I) Refinement

**Table:**

Refinement on *F*^2^	Primary atom site location: difference Fourier map
Least-squares matrix: full	Hydrogen site location: inferred from neighbouring sites
*R*[*F*^2^ > 2σ(*F*^2^)] = 0.055	H-atom parameters constrained
*wR*(*F*^2^) = 0.132	*w* = 1/[σ^2^(*F*_o_^2^) + (0.0322*P*)^2^ + 1.191*P*] where *P* = (*F*_o_^2^ + 2*F*_c_^2^)/3
*S* = 1.05	(Δ/σ)_max_ < 0.001
2912 reflections	Δρ_max_ = 0.32 e Å^−^^3^
190 parameters	Δρ_min_ = −0.23 e Å^−^^3^
0 restraints	

### 1-(4-Chlorophenyl)-3-[4-(prop-2-ynyloxy)phenyl]prop-2-en-1-one (I) Special details

**Table:**

Geometry. All esds (except the esd in the dihedral angle between two l.s. planes) are estimated using the full covariance matrix. The cell esds are taken into account individually in the estimation of esds in distances, angles and torsion angles; correlations between esds in cell parameters are only used when they are defined by crystal symmetry. An approximate (isotropic) treatment of cell esds is used for estimating esds involving l.s. planes.

### 1-(4-Chlorophenyl)-3-[4-(prop-2-ynyloxy)phenyl]prop-2-en-1-one (I) Fractional atomic coordinates and isotropic or equivalent isotropic displacement parameters (Å^2^)

**Table:**

	*x*	*y*	*z*	*U*_iso_*/*U*_eq_	
C1	0.50644 (15)	0.37616 (19)	0.0035 (5)	0.0454 (7)	
O1	0.49123 (11)	0.37815 (18)	−0.2039 (3)	0.0705 (7)	
C2	0.44832 (15)	0.37402 (19)	0.1650 (5)	0.0470 (7)	
H2	0.4612	0.3594	0.3175	0.056*	
C3	0.37764 (15)	0.39260 (17)	0.0980 (4)	0.0422 (6)	
H3	0.3682	0.4115	−0.0532	0.051*	
C11	0.58630 (14)	0.37539 (17)	0.0931 (4)	0.0388 (6)	
C12	0.60937 (15)	0.40870 (18)	0.3093 (4)	0.0443 (7)	
H12	0.5742	0.4301	0.4052	0.053*	
C13	0.68450 (16)	0.41034 (18)	0.3840 (5)	0.0464 (7)	
H13	0.7000	0.4340	0.5277	0.056*	
C14	0.73548 (14)	0.37664 (19)	0.2428 (5)	0.0455 (7)	
Cl14	0.82940 (4)	0.37534 (7)	0.33965 (16)	0.0789 (3)	
C15	0.71402 (16)	0.34316 (19)	0.0276 (5)	0.0506 (7)	
H15	0.7494	0.3208	−0.0665	0.061*	
C16	0.63951 (15)	0.34321 (18)	−0.0463 (5)	0.0463 (7)	
H16	0.6247	0.3213	−0.1921	0.056*	
C31	0.31322 (14)	0.38698 (17)	0.2325 (4)	0.0385 (6)	
C32	0.24378 (15)	0.41655 (19)	0.1399 (5)	0.0472 (7)	
H32	0.2398	0.4428	−0.0057	0.057*	
C33	0.18022 (16)	0.4082 (2)	0.2576 (5)	0.0542 (8)	
H33	0.1344	0.4289	0.1926	0.065*	
C34	0.18612 (15)	0.36873 (19)	0.4728 (5)	0.0478 (7)	
C35	0.25439 (15)	0.33836 (18)	0.5696 (5)	0.0460 (7)	
H35	0.2580	0.3117	0.7147	0.055*	
C36	0.31671 (15)	0.34757 (17)	0.4517 (4)	0.0429 (7)	
H36	0.3624	0.3272	0.5187	0.052*	
O34	0.12808 (12)	0.35634 (17)	0.6100 (4)	0.0737 (7)	
C37	0.05810 (19)	0.3821 (3)	0.5204 (6)	0.0851 (11)	
H37A	0.0428	0.3436	0.3886	0.102*	
H37B	0.0579	0.4473	0.4731	0.102*	
C38	0.0066 (2)	0.3679 (3)	0.7054 (7)	0.0920 (13)	
C39	−0.0365 (2)	0.3578 (4)	0.8370 (9)	0.1210 (18)	
H39	−0.0715	0.3496	0.9439	0.145*	

### 1-(4-Chlorophenyl)-3-[4-(prop-2-ynyloxy)phenyl]prop-2-en-1-one (I) Atomic displacement parameters (Å^2^)

**Table:**

	*U*^11^	*U*^22^	*U*^33^	*U*^12^	*U*^13^	*U*^23^
C1	0.0503 (16)	0.0486 (16)	0.0372 (16)	−0.0022 (13)	0.0016 (13)	0.0008 (13)
O1	0.0613 (14)	0.1132 (19)	0.0362 (12)	−0.0033 (13)	−0.0009 (10)	−0.0013 (12)
C2	0.0497 (17)	0.0545 (17)	0.0366 (15)	−0.0013 (14)	0.0012 (13)	0.0043 (13)
C3	0.0512 (16)	0.0407 (15)	0.0343 (15)	−0.0051 (12)	0.0010 (12)	0.0014 (12)
C11	0.0483 (16)	0.0374 (14)	0.0308 (14)	−0.0001 (12)	0.0041 (12)	0.0018 (12)
C12	0.0509 (17)	0.0470 (16)	0.0361 (16)	0.0048 (13)	0.0101 (13)	−0.0026 (12)
C13	0.0573 (18)	0.0476 (16)	0.0340 (15)	−0.0031 (13)	0.0018 (13)	−0.0036 (12)
C14	0.0445 (16)	0.0420 (15)	0.0496 (17)	−0.0016 (12)	0.0009 (13)	0.0031 (14)
Cl14	0.0491 (5)	0.0928 (7)	0.0938 (7)	−0.0010 (4)	−0.0009 (4)	−0.0102 (5)
C15	0.0517 (18)	0.0534 (17)	0.0487 (18)	−0.0009 (14)	0.0155 (14)	−0.0081 (14)
C16	0.0573 (18)	0.0478 (16)	0.0345 (15)	−0.0058 (13)	0.0086 (13)	−0.0037 (12)
C31	0.0479 (16)	0.0349 (14)	0.0320 (14)	−0.0017 (12)	−0.0011 (12)	−0.0018 (11)
C32	0.0562 (18)	0.0474 (16)	0.0373 (16)	0.0020 (14)	−0.0020 (14)	0.0041 (12)
C33	0.0449 (17)	0.0627 (19)	0.0540 (19)	0.0079 (14)	−0.0035 (14)	0.0037 (15)
C34	0.0476 (16)	0.0528 (17)	0.0435 (17)	−0.0019 (13)	0.0077 (13)	−0.0031 (14)
C35	0.0576 (18)	0.0475 (16)	0.0326 (15)	0.0032 (13)	0.0015 (13)	0.0022 (12)
C36	0.0466 (16)	0.0420 (15)	0.0392 (16)	0.0014 (12)	−0.0029 (13)	−0.0020 (12)
O34	0.0523 (13)	0.1029 (18)	0.0666 (15)	0.0097 (12)	0.0101 (11)	0.0078 (13)
C37	0.065 (2)	0.111 (3)	0.079 (3)	0.008 (2)	0.002 (2)	0.019 (2)
C38	0.050 (2)	0.128 (4)	0.099 (3)	−0.001 (2)	0.017 (2)	0.011 (3)
C39	0.062 (3)	0.189 (5)	0.115 (4)	0.007 (3)	0.021 (3)	0.034 (4)

### 1-(4-Chlorophenyl)-3-[4-(prop-2-ynyloxy)phenyl]prop-2-en-1-one (I) Geometric parameters (Å, º)

**Table:**

C1—O1	1.226 (3)	C31—C32	1.389 (3)
C1—C2	1.465 (4)	C31—C36	1.400 (3)
C1—C11	1.491 (4)	C32—C33	1.386 (4)
C2—C3	1.328 (3)	C32—H32	0.9300
C2—H2	0.9300	C33—C34	1.379 (4)
C3—C31	1.454 (4)	C33—H33	0.9300
C3—H3	0.9300	C34—O34	1.378 (3)
C11—C16	1.385 (3)	C34—C35	1.382 (4)
C11—C12	1.387 (3)	C35—C36	1.368 (4)
C12—C13	1.388 (4)	C35—H35	0.9300
C12—H12	0.9300	C36—H36	0.9300
C13—C14	1.369 (4)	O34—C37	1.376 (4)
C13—H13	0.9300	C37—C38	1.494 (5)
C14—C15	1.377 (4)	C37—H37A	0.9700
C14—Cl14	1.741 (3)	C37—H37B	0.9700
C15—C16	1.377 (4)	C38—C39	1.144 (5)
C15—H15	0.9300	C39—H39	0.9300
C16—H16	0.9300		
			
O1—C1—C2	121.8 (3)	C32—C31—C36	117.2 (2)
O1—C1—C11	119.0 (2)	C32—C31—C3	120.1 (2)
C2—C1—C11	119.2 (2)	C36—C31—C3	122.6 (2)
C3—C2—C1	121.3 (2)	C33—C32—C31	122.1 (3)
C3—C2—H2	119.4	C33—C32—H32	119.0
C1—C2—H2	119.4	C31—C32—H32	119.0
C2—C3—C31	127.8 (2)	C34—C33—C32	118.9 (3)
C2—C3—H3	116.1	C34—C33—H33	120.5
C31—C3—H3	116.1	C32—C33—H33	120.5
C16—C11—C12	118.8 (2)	O34—C34—C33	125.4 (3)
C16—C11—C1	119.0 (2)	O34—C34—C35	114.2 (2)
C12—C11—C1	122.2 (2)	C33—C34—C35	120.5 (3)
C11—C12—C13	120.6 (2)	C36—C35—C34	119.9 (3)
C11—C12—H12	119.7	C36—C35—H35	120.0
C13—C12—H12	119.7	C34—C35—H35	120.0
C14—C13—C12	119.0 (3)	C35—C36—C31	121.5 (3)
C14—C13—H13	120.5	C35—C36—H36	119.3
C12—C13—H13	120.5	C31—C36—H36	119.3
C13—C14—C15	121.5 (3)	C37—O34—C34	117.1 (3)
C13—C14—Cl14	119.0 (2)	O34—C37—C38	106.7 (3)
C15—C14—Cl14	119.5 (2)	O34—C37—H37A	110.4
C16—C15—C14	119.1 (3)	C38—C37—H37A	110.4
C16—C15—H15	120.5	O34—C37—H37B	110.4
C14—C15—H15	120.5	C38—C37—H37B	110.4
C15—C16—C11	121.0 (2)	H37A—C37—H37B	108.6
C15—C16—H16	119.5	C39—C38—C37	175.7 (5)
C11—C16—H16	119.5	C38—C39—H39	180.0
			
O1—C1—C2—C3	−13.4 (4)	C1—C11—C16—C15	178.6 (2)
C11—C1—C2—C3	166.9 (2)	C2—C3—C31—C32	174.7 (3)
C1—C2—C3—C31	175.3 (2)	C2—C3—C31—C36	−9.0 (4)
O1—C1—C11—C16	−23.3 (4)	C36—C31—C32—C33	0.2 (4)
C2—C1—C11—C16	156.4 (2)	C3—C31—C32—C33	176.8 (2)
O1—C1—C11—C12	154.6 (3)	C31—C32—C33—C34	−0.4 (4)
C2—C1—C11—C12	−25.7 (4)	C32—C33—C34—O34	179.7 (3)
C16—C11—C12—C13	0.4 (4)	C32—C33—C34—C35	0.2 (4)
C1—C11—C12—C13	−177.5 (2)	O34—C34—C35—C36	−179.4 (2)
C11—C12—C13—C14	−1.4 (4)	C33—C34—C35—C36	0.1 (4)
C12—C13—C14—C15	1.4 (4)	C34—C35—C36—C31	−0.3 (4)
C12—C13—C14—Cl14	−178.0 (2)	C32—C31—C36—C35	0.1 (4)
C13—C14—C15—C16	−0.4 (4)	C3—C31—C36—C35	−176.3 (2)
Cl14—C14—C15—C16	179.1 (2)	C33—C34—O34—C37	2.8 (4)
C14—C15—C16—C11	−0.7 (4)	C35—C34—O34—C37	−177.8 (3)
C12—C11—C16—C15	0.6 (4)	C34—O34—C37—C38	−176.4 (3)

### 1-(4-Chlorophenyl)-3-[4-(prop-2-ynyloxy)phenyl]prop-2-en-1-one (I) Hydrogen-bond geometry (Å, º)

**Table:**

*D*—H···*A*	*D*—H	H···*A*	*D*···*A*	*D*—H···*A*
C13—H13···*Cg*1^i^	0.93	2.90	3.554 (3)	128
C35—H35···*Cg*1^ii^	0.93	2.83	3.508 (3)	131

Symmetry codes: (i) −*x*+1, −*y*+1, −*z*+1; (ii) *x*, −*y*+1/2, *z*+1/2.

### 1-(4-Bromophenyl)-3-[4-(prop-2-ynyloxy)phenyl]prop-2-en-1-one (II) Crystal data

**Table:**

C_18_H_13_BrO_2_	*F*(000) = 688
*M**_r_* = 341.18	*D*_x_ = 1.489 Mg m^−^^3^
Monoclinic, *P*2_1_/*c*	Mo *K*α radiation, λ = 0.71073 Å
*a* = 18.286 (6) Å	Cell parameters from 2047 reflections
*b* = 14.277 (4) Å	θ = 1.1–26.2°
*c* = 5.8489 (17) Å	µ = 2.70 mm^−^^1^
β = 94.521 (7)°	*T* = 296 K
*V* = 1522.2 (8) Å^3^	Block, colourless
*Z* = 4	0.20 × 0.15 × 0.15 mm

### 1-(4-Bromophenyl)-3-[4-(prop-2-ynyloxy)phenyl]prop-2-en-1-one (II) Data collection

**Table:**

Bruker APEXII diffractometer	2945 independent reflections
Radiation source: fine focussealed tube	1335 reflections with *I* > 2σ(*I*)
Graphite monochromator	*R*_int_ = 0.119
φ and ω scans	θ_max_ = 26.2°, θ_min_ = 2.2°
Absorption correction: multi-scan (SADABS; Bruker, 2012)	*h* = −22→22
*T*_min_ = 0.491, *T*_max_ = 0.667	*k* = −17→17
23006 measured reflections	*l* = −7→7

### 1-(4-Bromophenyl)-3-[4-(prop-2-ynyloxy)phenyl]prop-2-en-1-one (II) Refinement

**Table:**

Refinement on *F*^2^	Primary atom site location: difference Fourier map
Least-squares matrix: full	Hydrogen site location: inferred from neighbouring sites
*R*[*F*^2^ > 2σ(*F*^2^)] = 0.043	H-atom parameters constrained
*wR*(*F*^2^) = 0.089	*w* = 1/[σ^2^(*F*_o_^2^) + (0.0262*P*)^2^ + 0.1591*P*] where *P* = (*F*_o_^2^ + 2*F*_c_^2^)/3
*S* = 1.00	(Δ/σ)_max_ = 0.001
2945 reflections	Δρ_max_ = 0.43 e Å^−^^3^
190 parameters	Δρ_min_ = −0.50 e Å^−^^3^
0 restraints	

### 1-(4-Bromophenyl)-3-[4-(prop-2-ynyloxy)phenyl]prop-2-en-1-one (II) Special details

**Table:**

Geometry. All esds (except the esd in the dihedral angle between two l.s. planes) are estimated using the full covariance matrix. The cell esds are taken into account individually in the estimation of esds in distances, angles and torsion angles; correlations between esds in cell parameters are only used when they are defined by crystal symmetry. An approximate (isotropic) treatment of cell esds is used for estimating esds involving l.s. planes.

### 1-(4-Bromophenyl)-3-[4-(prop-2-ynyloxy)phenyl]prop-2-en-1-one (II) Fractional atomic coordinates and isotropic or equivalent isotropic displacement parameters (Å^2^)

**Table:**

	*x*	*y*	*z*	*U*_iso_*/*U*_eq_	
C1	0.5049 (2)	0.3768 (3)	0.0000 (7)	0.0455 (10)	
O1	0.48975 (14)	0.3791 (2)	−0.2076 (4)	0.0658 (8)	
C2	0.4477 (2)	0.3747 (3)	0.1628 (6)	0.0428 (10)	
H2	0.4606	0.3605	0.3158	0.051*	
C3	0.3782 (2)	0.3926 (2)	0.0970 (6)	0.0398 (10)	
H3	0.3689	0.4117	−0.0545	0.048*	
C11	0.5835 (2)	0.3755 (2)	0.0928 (6)	0.0355 (9)	
C12	0.6067 (2)	0.4089 (2)	0.3083 (6)	0.0420 (11)	
H12	0.5723	0.4309	0.4041	0.050*	
C13	0.6798 (2)	0.4103 (2)	0.3835 (6)	0.0433 (11)	
H13	0.6948	0.4337	0.5281	0.052*	
C14	0.7306 (2)	0.3767 (3)	0.2425 (6)	0.0431 (10)	
Br14	0.83135 (2)	0.37416 (4)	0.34974 (8)	0.0703 (2)	
C15	0.7097 (2)	0.3428 (2)	0.0268 (7)	0.0458 (11)	
H15	0.7444	0.3200	−0.0668	0.055*	
C16	0.6364 (2)	0.3435 (2)	−0.0474 (6)	0.0444 (11)	
H16	0.6218	0.3223	−0.1942	0.053*	
C31	0.3145 (2)	0.3864 (2)	0.2299 (6)	0.0350 (9)	
C32	0.2465 (2)	0.4154 (2)	0.1353 (6)	0.0448 (11)	
H32	0.2433	0.4417	−0.0107	0.054*	
C33	0.1829 (2)	0.4070 (3)	0.2489 (7)	0.0472 (11)	
H33	0.1379	0.4273	0.1818	0.057*	
C34	0.1890 (2)	0.3672 (3)	0.4658 (6)	0.0433 (10)	
C35	0.2557 (2)	0.3374 (2)	0.5652 (6)	0.0404 (10)	
H35	0.2588	0.3107	0.7107	0.049*	
C36	0.3175 (2)	0.3473 (2)	0.4488 (6)	0.0390 (10)	
H36	0.3624	0.3275	0.5175	0.047*	
O34	0.13136 (16)	0.35477 (19)	0.6007 (4)	0.0638 (8)	
C37	0.0619 (2)	0.3799 (3)	0.5085 (7)	0.0726 (13)	
H37A	0.0474	0.3410	0.3766	0.087*	
H37B	0.0616	0.4448	0.4596	0.087*	
C38	0.0106 (3)	0.3664 (4)	0.6897 (9)	0.0806 (15)	
C39	−0.0320 (3)	0.3582 (4)	0.8233 (9)	0.106 (2)	
H39	−0.0664	0.3516	0.9312	0.127*	

### 1-(4-Bromophenyl)-3-[4-(prop-2-ynyloxy)phenyl]prop-2-en-1-one (II) Atomic displacement parameters (Å^2^)

**Table:**

	*U*^11^	*U*^22^	*U*^33^	*U*^12^	*U*^13^	*U*^23^
C1	0.050 (3)	0.045 (3)	0.041 (3)	−0.001 (2)	0.003 (2)	0.002 (2)
O1	0.058 (2)	0.110 (2)	0.0289 (16)	−0.0013 (18)	−0.0015 (14)	−0.0012 (17)
C2	0.044 (3)	0.050 (2)	0.034 (2)	−0.005 (2)	−0.002 (2)	0.004 (2)
C3	0.051 (3)	0.035 (3)	0.032 (2)	−0.008 (2)	−0.002 (2)	0.0031 (18)
C11	0.044 (3)	0.033 (2)	0.030 (2)	0.002 (2)	0.005 (2)	0.004 (2)
C12	0.048 (3)	0.046 (3)	0.032 (2)	0.006 (2)	0.007 (2)	−0.0007 (18)
C13	0.054 (3)	0.042 (3)	0.034 (2)	−0.003 (2)	0.000 (2)	−0.0056 (18)
C14	0.040 (3)	0.039 (2)	0.050 (3)	−0.001 (2)	0.001 (2)	0.001 (2)
Br14	0.0455 (3)	0.0781 (3)	0.0860 (4)	−0.0015 (3)	−0.0030 (2)	−0.0081 (3)
C15	0.045 (3)	0.047 (3)	0.048 (3)	0.001 (2)	0.014 (2)	−0.010 (2)
C16	0.058 (3)	0.043 (3)	0.032 (2)	−0.005 (2)	0.003 (2)	−0.0052 (18)
C31	0.044 (3)	0.033 (2)	0.028 (2)	−0.001 (2)	0.000 (2)	−0.0026 (19)
C32	0.051 (3)	0.047 (3)	0.034 (2)	0.006 (2)	−0.008 (2)	0.0047 (18)
C33	0.037 (3)	0.057 (3)	0.047 (3)	0.010 (2)	−0.001 (2)	0.004 (2)
C34	0.046 (3)	0.046 (2)	0.039 (2)	−0.001 (2)	0.009 (2)	−0.005 (2)
C35	0.048 (3)	0.041 (3)	0.031 (2)	0.001 (2)	0.000 (2)	0.0030 (18)
C36	0.038 (3)	0.040 (3)	0.037 (2)	−0.0017 (19)	−0.006 (2)	−0.0015 (18)
O34	0.0415 (19)	0.091 (2)	0.0588 (19)	0.0127 (17)	0.0053 (16)	0.0078 (16)
C37	0.060 (4)	0.088 (4)	0.070 (3)	0.009 (3)	0.000 (3)	0.008 (3)
C38	0.047 (4)	0.109 (4)	0.086 (4)	0.003 (4)	0.009 (3)	0.004 (4)
C39	0.065 (4)	0.162 (6)	0.093 (4)	0.014 (4)	0.024 (3)	0.023 (4)

### 1-(4-Bromophenyl)-3-[4-(prop-2-ynyloxy)phenyl]prop-2-en-1-one (II) Geometric parameters (Å, º)

**Table:**

C1—O1	1.225 (4)	C31—C32	1.383 (5)
C1—C2	1.470 (5)	C31—C36	1.394 (5)
C1—C11	1.496 (5)	C32—C33	1.390 (5)
C2—C3	1.323 (5)	C32—H32	0.9300
C2—H2	0.9300	C33—C34	1.386 (5)
C3—C31	1.453 (5)	C33—H33	0.9300
C3—H3	0.9300	C34—C35	1.376 (5)
C11—C12	1.383 (5)	C34—O34	1.377 (4)
C11—C16	1.393 (5)	C35—C36	1.373 (5)
C12—C13	1.373 (5)	C35—H35	0.9300
C12—H12	0.9300	C36—H36	0.9300
C13—C14	1.376 (5)	O34—C37	1.388 (4)
C13—H13	0.9300	C37—C38	1.483 (6)
C14—C15	1.377 (5)	C37—H37A	0.9700
C14—Br14	1.899 (4)	C37—H37B	0.9700
C15—C16	1.376 (5)	C38—C39	1.152 (6)
C15—H15	0.9300	C39—H39	0.9300
C16—H16	0.9300		
			
O1—C1—C2	121.7 (4)	C32—C31—C36	117.1 (3)
O1—C1—C11	119.8 (3)	C32—C31—C3	120.0 (3)
C2—C1—C11	118.5 (3)	C36—C31—C3	122.7 (4)
C3—C2—C1	121.6 (3)	C31—C32—C33	122.8 (4)
C3—C2—H2	119.2	C31—C32—H32	118.6
C1—C2—H2	119.2	C33—C32—H32	118.6
C2—C3—C31	128.6 (3)	C34—C33—C32	117.6 (4)
C2—C3—H3	115.7	C34—C33—H33	121.2
C31—C3—H3	115.7	C32—C33—H33	121.2
C12—C11—C16	118.1 (4)	C35—C34—O34	114.2 (3)
C12—C11—C1	123.0 (3)	C35—C34—C33	121.1 (4)
C16—C11—C1	118.8 (3)	O34—C34—C33	124.6 (4)
C13—C12—C11	121.2 (3)	C36—C35—C34	119.7 (3)
C13—C12—H12	119.4	C36—C35—H35	120.1
C11—C12—H12	119.4	C34—C35—H35	120.1
C12—C13—C14	119.3 (3)	C35—C36—C31	121.5 (4)
C12—C13—H13	120.4	C35—C36—H36	119.2
C14—C13—H13	120.4	C31—C36—H36	119.2
C13—C14—C15	121.3 (4)	C34—O34—C37	117.5 (3)
C13—C14—Br14	119.5 (3)	O34—C37—C38	107.5 (4)
C15—C14—Br14	119.2 (3)	O34—C37—H37A	110.2
C16—C15—C14	118.7 (3)	C38—C37—H37A	110.2
C16—C15—H15	120.7	O34—C37—H37B	110.2
C14—C15—H15	120.7	C38—C37—H37B	110.2
C15—C16—C11	121.4 (3)	H37A—C37—H37B	108.5
C15—C16—H16	119.3	C39—C38—C37	176.5 (6)
C11—C16—H16	119.3	C38—C39—H39	180.0
			
O1—C1—C2—C3	−12.7 (6)	C1—C11—C16—C15	178.4 (3)
C11—C1—C2—C3	167.6 (4)	C2—C3—C31—C32	174.8 (4)
C1—C2—C3—C31	174.6 (3)	C2—C3—C31—C36	−8.7 (6)
O1—C1—C11—C12	153.8 (4)	C36—C31—C32—C33	0.0 (5)
C2—C1—C11—C12	−26.5 (6)	C3—C31—C32—C33	176.7 (3)
O1—C1—C11—C16	−22.9 (6)	C31—C32—C33—C34	−0.3 (6)
C2—C1—C11—C16	156.8 (3)	C32—C33—C34—C35	0.2 (6)
C16—C11—C12—C13	−0.4 (5)	C32—C33—C34—O34	179.4 (3)
C1—C11—C12—C13	−177.1 (3)	O34—C34—C35—C36	−179.1 (3)
C11—C12—C13—C14	−0.8 (5)	C33—C34—C35—C36	0.2 (6)
C12—C13—C14—C15	0.8 (5)	C34—C35—C36—C31	−0.5 (5)
C12—C13—C14—Br14	−177.7 (3)	C32—C31—C36—C35	0.4 (5)
C13—C14—C15—C16	0.4 (5)	C3—C31—C36—C35	−176.2 (3)
Br14—C14—C15—C16	178.9 (3)	C35—C34—O34—C37	−177.6 (3)
C14—C15—C16—C11	−1.6 (5)	C33—C34—O34—C37	3.1 (6)
C12—C11—C16—C15	1.6 (5)	C34—O34—C37—C38	−176.1 (3)

### 1-(4-Bromophenyl)-3-[4-(prop-2-ynyloxy)phenyl]prop-2-en-1-one (II) Hydrogen-bond geometry (Å, º)

**Table:**

*D*—H···*A*	*D*—H	H···*A*	*D*···*A*	*D*—H···*A*
C13—H13···*Cg*1^i^	0.93	2.95	3.602 (4)	128
C35—H35···*Cg*1^ii^	0.93	2.80	3.484 (3)	131

Symmetry codes: (i) −*x*+1, −*y*+1, −*z*+1; (ii) *x*, −*y*+1/2, *z*+1/2.

### (RS)-3-(4-Chlorophenyl)-5-[4-(prop-2-ynyloxy)phenyl]-4,5-dihydropyrazole-1-carbothioamide (IV) Crystal data

**Table:**

C_19_H_16_ClN_3_OS	*F*(000) = 768
*M**_r_* = 369.86	*D*_x_ = 1.385 Mg m^−^^3^
Monoclinic, *P*2_1_/*n*	Mo *K*α radiation, λ = 0.71073 Å
*a* = 15.0182 (9) Å	Cell parameters from 4932 reflections
*b* = 6.0579 (3) Å	θ = 2.9–29.5°
*c* = 20.8286 (12) Å	µ = 0.35 mm^−^^1^
β = 110.573 (2)°	*T* = 298 K
*V* = 1774.11 (17) Å^3^	Needle, colourless
*Z* = 4	0.20 × 0.15 × 0.10 mm

### (RS)-3-(4-Chlorophenyl)-5-[4-(prop-2-ynyloxy)phenyl]-4,5-dihydropyrazole-1-carbothioamide (IV) Data collection

**Table:**

Bruker APEXII diffractometer	3326 independent reflections
Radiation source: fine focussealed tube	2571 reflections with *I* > 2σ(*I*)
Graphite monochromator	*R*_int_ = 0.064
φ and ω scans	θ_max_ = 25.6°, θ_min_ = 3.5°
Absorption correction: multi-scan (SADABS; Bruker, 2012)	*h* = −18→18
*T*_min_ = 0.870, *T*_max_ = 0.966	*k* = −7→7
25833 measured reflections	*l* = −25→25

### (RS)-3-(4-Chlorophenyl)-5-[4-(prop-2-ynyloxy)phenyl]-4,5-dihydropyrazole-1-carbothioamide (IV) Refinement

**Table:**

Refinement on *F*^2^	Primary atom site location: difference Fourier map
Least-squares matrix: full	Hydrogen site location: mixed
*R*[*F*^2^ > 2σ(*F*^2^)] = 0.075	H atoms treated by a mixture of independent and constrained refinement
*wR*(*F*^2^) = 0.133	*w* = 1/[σ^2^(*F*_o_^2^) + (0.0304*P*)^2^ + 1.7629*P*] where *P* = (*F*_o_^2^ + 2*F*_c_^2^)/3
*S* = 1.24	(Δ/σ)_max_ < 0.001
3326 reflections	Δρ_max_ = 0.19 e Å^−^^3^
232 parameters	Δρ_min_ = −0.28 e Å^−^^3^
0 restraints	

### (RS)-3-(4-Chlorophenyl)-5-[4-(prop-2-ynyloxy)phenyl]-4,5-dihydropyrazole-1-carbothioamide (IV) Special details

**Table:**

Geometry. All esds (except the esd in the dihedral angle between two l.s. planes) are estimated using the full covariance matrix. The cell esds are taken into account individually in the estimation of esds in distances, angles and torsion angles; correlations between esds in cell parameters are only used when they are defined by crystal symmetry. An approximate (isotropic) treatment of cell esds is used for estimating esds involving l.s. planes.

### (RS)-3-(4-Chlorophenyl)-5-[4-(prop-2-ynyloxy)phenyl]-4,5-dihydropyrazole-1-carbothioamide (IV) Fractional atomic coordinates and isotropic or equivalent isotropic displacement parameters (Å^2^)

**Table:**

	*x*	*y*	*z*	*U*_iso_*/*U*_eq_	
N1	0.5173 (2)	0.7308 (4)	0.34225 (14)	0.0412 (7)	
N2	0.45647 (18)	0.5533 (4)	0.31707 (14)	0.0410 (7)	
C3	0.4545 (2)	0.5106 (6)	0.25593 (17)	0.0409 (8)	
C4	0.5122 (2)	0.6700 (6)	0.23130 (17)	0.0458 (9)	
H4A	0.4717	0.7751	0.1990	0.055*	
H4	0.5513	0.5935	0.2100	0.055*	
C5	0.5734 (2)	0.7840 (6)	0.29827 (17)	0.0427 (8)	
H5	0.5753	0.9439	0.2916	0.051*	
C11	0.5230 (2)	0.8250 (6)	0.40229 (17)	0.0402 (8)	
S11	0.59292 (7)	1.04457 (15)	0.43338 (5)	0.0499 (3)	
N11	0.4696 (3)	0.7346 (6)	0.43395 (18)	0.0623 (10)	
H11B	0.466 (3)	0.803 (7)	0.470 (2)	0.075*	
H11A	0.439 (3)	0.628 (7)	0.417 (2)	0.075*	
C31	0.3970 (2)	0.3293 (6)	0.21696 (17)	0.0411 (8)	
C32	0.3691 (3)	0.3240 (7)	0.14614 (19)	0.0619 (11)	
H32	0.3909	0.4320	0.1236	0.074*	
C33	0.3095 (3)	0.1608 (8)	0.1088 (2)	0.0668 (12)	
H33	0.2907	0.1593	0.0612	0.080*	
C34	0.2781 (2)	0.0015 (6)	0.1419 (2)	0.0531 (10)	
Cl34	0.20074 (7)	−0.2014 (2)	0.09434 (6)	0.0773 (4)	
C35	0.3074 (3)	−0.0024 (6)	0.2126 (2)	0.0535 (10)	
H35	0.2874	−0.1146	0.2348	0.064*	
C36	0.3663 (2)	0.1615 (6)	0.24951 (19)	0.0475 (9)	
H36	0.3861	0.1600	0.2971	0.057*	
C51	0.6726 (2)	0.6900 (5)	0.32626 (16)	0.0397 (8)	
C52	0.6902 (3)	0.4924 (6)	0.36169 (18)	0.0497 (9)	
H52	0.6404	0.4210	0.3698	0.060*	
C53	0.7795 (2)	0.3978 (6)	0.38544 (18)	0.0468 (9)	
H53	0.7896	0.2643	0.4090	0.056*	
C54	0.8535 (2)	0.5047 (6)	0.37368 (16)	0.0417 (8)	
C55	0.8367 (3)	0.6981 (6)	0.33696 (17)	0.0457 (9)	
H55	0.8863	0.7672	0.3278	0.055*	
C56	0.7476 (3)	0.7905 (6)	0.31355 (17)	0.0436 (8)	
H56	0.7374	0.9219	0.2889	0.052*	
O54	0.94624 (17)	0.4330 (4)	0.39776 (13)	0.0540 (7)	
C57	0.9668 (3)	0.2523 (6)	0.4444 (2)	0.0563 (10)	
H57A	0.9342	0.1209	0.4211	0.068*	
H57B	0.9446	0.2847	0.4818	0.068*	
C58	1.0686 (3)	0.2143 (6)	0.47095 (19)	0.0526 (9)	
C59	1.1497 (3)	0.1775 (7)	0.4949 (2)	0.0631 (11)	
H59	1.2145	0.1481	0.5141	0.076*	

### (RS)-3-(4-Chlorophenyl)-5-[4-(prop-2-ynyloxy)phenyl]-4,5-dihydropyrazole-1-carbothioamide (IV) Atomic displacement parameters (Å^2^)

**Table:**

	*U*^11^	*U*^22^	*U*^33^	*U*^12^	*U*^13^	*U*^23^
N1	0.0464 (16)	0.0399 (16)	0.0436 (16)	−0.0078 (14)	0.0237 (13)	−0.0016 (13)
N2	0.0408 (16)	0.0402 (16)	0.0450 (16)	−0.0051 (14)	0.0186 (13)	0.0003 (14)
C3	0.0390 (19)	0.043 (2)	0.0432 (19)	0.0045 (16)	0.0169 (16)	0.0046 (16)
C4	0.048 (2)	0.050 (2)	0.0420 (19)	0.0046 (18)	0.0192 (17)	0.0044 (17)
C5	0.052 (2)	0.0373 (19)	0.047 (2)	−0.0016 (17)	0.0278 (17)	0.0068 (16)
C11	0.0413 (19)	0.0395 (19)	0.0426 (19)	0.0000 (16)	0.0181 (16)	0.0028 (16)
S11	0.0589 (6)	0.0428 (5)	0.0537 (6)	−0.0133 (5)	0.0268 (5)	−0.0037 (5)
N11	0.079 (3)	0.068 (2)	0.057 (2)	−0.0352 (19)	0.044 (2)	−0.0192 (18)
C31	0.0377 (19)	0.0428 (19)	0.0401 (19)	0.0067 (16)	0.0103 (15)	0.0007 (16)
C32	0.072 (3)	0.066 (3)	0.045 (2)	−0.010 (2)	0.017 (2)	0.001 (2)
C33	0.073 (3)	0.076 (3)	0.044 (2)	−0.003 (3)	0.013 (2)	−0.011 (2)
C34	0.038 (2)	0.055 (2)	0.064 (3)	0.0043 (18)	0.0144 (19)	−0.016 (2)
Cl34	0.0552 (6)	0.0823 (8)	0.0933 (8)	−0.0106 (6)	0.0247 (6)	−0.0430 (7)
C35	0.051 (2)	0.047 (2)	0.066 (3)	0.0004 (18)	0.025 (2)	−0.0029 (19)
C36	0.050 (2)	0.047 (2)	0.046 (2)	0.0060 (18)	0.0171 (17)	0.0010 (18)
C51	0.047 (2)	0.0379 (19)	0.0382 (18)	−0.0054 (16)	0.0199 (16)	0.0000 (15)
C52	0.049 (2)	0.044 (2)	0.063 (2)	−0.0090 (18)	0.0285 (19)	0.0071 (18)
C53	0.049 (2)	0.0379 (19)	0.057 (2)	−0.0033 (17)	0.0227 (18)	0.0087 (17)
C54	0.042 (2)	0.046 (2)	0.0390 (18)	−0.0056 (17)	0.0177 (16)	−0.0067 (16)
C55	0.048 (2)	0.050 (2)	0.047 (2)	−0.0132 (18)	0.0275 (17)	0.0027 (18)
C56	0.055 (2)	0.0383 (19)	0.0429 (19)	−0.0081 (17)	0.0241 (17)	0.0071 (16)
O54	0.0466 (15)	0.0592 (16)	0.0609 (16)	0.0010 (13)	0.0247 (13)	0.0083 (14)
C57	0.056 (2)	0.056 (2)	0.058 (2)	0.0017 (19)	0.022 (2)	−0.003 (2)
C58	0.055 (3)	0.056 (2)	0.049 (2)	−0.001 (2)	0.021 (2)	−0.0060 (19)
C59	0.058 (3)	0.078 (3)	0.053 (2)	0.002 (2)	0.019 (2)	0.001 (2)

### (RS)-3-(4-Chlorophenyl)-5-[4-(prop-2-ynyloxy)phenyl]-4,5-dihydropyrazole-1-carbothioamide (IV) Geometric parameters (Å, º)

**Table:**

N1—C11	1.350 (4)	C34—Cl34	1.741 (4)
N1—N2	1.389 (4)	C35—C36	1.372 (5)
N1—C5	1.481 (4)	C35—H35	0.9300
N2—C3	1.290 (4)	C36—H36	0.9300
C3—C31	1.456 (5)	C51—C52	1.382 (5)
C3—C4	1.502 (5)	C51—C56	1.385 (4)
C4—C5	1.539 (5)	C52—C53	1.380 (5)
C4—H4A	0.9700	C52—H52	0.9300
C4—H4	0.9700	C53—C54	1.380 (4)
C5—C51	1.507 (5)	C53—H53	0.9300
C5—H5	0.9800	C54—C55	1.373 (5)
C11—N11	1.323 (4)	C54—O54	1.375 (4)
C11—S11	1.677 (3)	C55—C56	1.373 (5)
N11—H11B	0.87 (4)	C55—H55	0.9300
N11—H11A	0.80 (4)	C56—H56	0.9300
C31—C32	1.385 (5)	O54—C57	1.423 (4)
C31—C36	1.387 (5)	C57—C58	1.450 (5)
C32—C33	1.376 (6)	C57—H57A	0.9700
C32—H32	0.9300	C57—H57B	0.9700
C33—C34	1.364 (6)	C58—C59	1.164 (5)
C33—H33	0.9300	C59—H59	0.9300
C34—C35	1.380 (5)		
			
C11—N1—N2	119.7 (3)	C33—C34—Cl34	119.5 (3)
C11—N1—C5	128.1 (3)	C35—C34—Cl34	119.6 (3)
N2—N1—C5	112.1 (2)	C36—C35—C34	119.1 (4)
C3—N2—N1	108.1 (3)	C36—C35—H35	120.4
N2—C3—C31	120.3 (3)	C34—C35—H35	120.4
N2—C3—C4	113.1 (3)	C35—C36—C31	121.0 (3)
C31—C3—C4	126.5 (3)	C35—C36—H36	119.5
C3—C4—C5	102.2 (3)	C31—C36—H36	119.5
C3—C4—H4A	111.3	C52—C51—C56	117.8 (3)
C5—C4—H4A	111.3	C52—C51—C5	120.8 (3)
C3—C4—H4	111.3	C56—C51—C5	121.3 (3)
C5—C4—H4	111.3	C53—C52—C51	122.1 (3)
H4A—C4—H4	109.2	C53—C52—H52	119.0
N1—C5—C51	112.2 (3)	C51—C52—H52	119.0
N1—C5—C4	100.0 (3)	C54—C53—C52	118.9 (3)
C51—C5—C4	112.1 (3)	C54—C53—H53	120.6
N1—C5—H5	110.7	C52—C53—H53	120.6
C51—C5—H5	110.7	C55—C54—O54	115.9 (3)
C4—C5—H5	110.7	C55—C54—C53	119.9 (3)
N11—C11—N1	115.8 (3)	O54—C54—C53	124.2 (3)
N11—C11—S11	122.9 (3)	C54—C55—C56	120.7 (3)
N1—C11—S11	121.3 (2)	C54—C55—H55	119.7
C11—N11—H11B	117 (3)	C56—C55—H55	119.7
C11—N11—H11A	118 (3)	C55—C56—C51	120.7 (3)
H11B—N11—H11A	124 (4)	C55—C56—H56	119.7
C32—C31—C36	118.4 (3)	C51—C56—H56	119.7
C32—C31—C3	120.6 (3)	C54—O54—C57	116.2 (3)
C36—C31—C3	121.0 (3)	O54—C57—C58	109.2 (3)
C33—C32—C31	120.8 (4)	O54—C57—H57A	109.8
C33—C32—H32	119.6	C58—C57—H57A	109.8
C31—C32—H32	119.6	O54—C57—H57B	109.8
C34—C33—C32	119.6 (4)	C58—C57—H57B	109.8
C34—C33—H33	120.2	H57A—C57—H57B	108.3
C32—C33—H33	120.2	C59—C58—C57	176.5 (4)
C33—C34—C35	121.0 (4)	C58—C59—H59	180.0
			
C11—N1—N2—C3	172.1 (3)	C32—C33—C34—Cl34	178.7 (3)
C5—N1—N2—C3	−11.1 (4)	C33—C34—C35—C36	2.3 (5)
N1—N2—C3—C31	179.3 (3)	Cl34—C34—C35—C36	−178.3 (3)
N1—N2—C3—C4	−3.4 (4)	C34—C35—C36—C31	−0.2 (5)
N2—C3—C4—C5	15.3 (4)	C32—C31—C36—C35	−2.1 (5)
C31—C3—C4—C5	−167.6 (3)	C3—C31—C36—C35	176.1 (3)
C11—N1—C5—C51	77.1 (4)	N1—C5—C51—C52	32.9 (4)
N2—N1—C5—C51	−99.5 (3)	C4—C5—C51—C52	−78.7 (4)
C11—N1—C5—C4	−164.0 (3)	N1—C5—C51—C56	−151.4 (3)
N2—N1—C5—C4	19.5 (3)	C4—C5—C51—C56	97.0 (4)
C3—C4—C5—N1	−19.2 (3)	C56—C51—C52—C53	1.4 (5)
C3—C4—C5—C51	99.8 (3)	C5—C51—C52—C53	177.3 (3)
N2—N1—C11—N11	1.3 (5)	C51—C52—C53—C54	0.3 (5)
C5—N1—C11—N11	−175.0 (3)	C52—C53—C54—C55	−2.1 (5)
N2—N1—C11—S11	−178.0 (2)	C52—C53—C54—O54	176.5 (3)
C5—N1—C11—S11	5.6 (5)	O54—C54—C55—C56	−176.7 (3)
N2—C3—C31—C32	158.8 (3)	C53—C54—C55—C56	2.1 (5)
C4—C3—C31—C32	−18.1 (5)	C54—C55—C56—C51	−0.3 (5)
N2—C3—C31—C36	−19.3 (5)	C52—C51—C56—C55	−1.5 (5)
C4—C3—C31—C36	163.8 (3)	C5—C51—C56—C55	−177.3 (3)
C36—C31—C32—C33	2.5 (6)	C55—C54—O54—C57	170.8 (3)
C3—C31—C32—C33	−175.7 (4)	C53—C54—O54—C57	−7.9 (5)
C31—C32—C33—C34	−0.5 (6)	C54—O54—C57—C58	−173.3 (3)
C32—C33—C34—C35	−1.9 (6)		

### (RS)-3-(4-Chlorophenyl)-5-[4-(prop-2-ynyloxy)phenyl]-4,5-dihydropyrazole-1-carbothioamide (IV) Hydrogen-bond geometry (Å, º)

**Table:**

*D*—H···*A*	*D*—H	H···*A*	*D*···*A*	*D*—H···*A*
N11—H11*A*···N2	0.80 (4)	2.23 (4)	2.614 (5)	110 (4)
N11—H11*B*···S11^i^	0.88 (4)	2.63 (4)	3.483 (4)	164 (4)
C52—H52···S11^ii^	0.93	2.85	3.641 (4)	144

Symmetry codes: (i) −*x*+1, −*y*+2, −*z*+1; (ii) *x*, *y*−1, *z*.

### (RS)-3-(4-Bromophenyl)-5-[4-(prop-2-ynyloxy)phenyl]-4,5-dihydropyrazole-1-carbothioamide (V) Crystal data

**Table:**

C_19_H_16_BrN_3_OS	*F*(000) = 840
*M**_r_* = 414.31	*D*_x_ = 1.529 Mg m^−^^3^
Monoclinic, *P*2_1_/*n*	Mo *K*α radiation, λ = 0.71073 Å
*a* = 15.1255 (13) Å	Cell parameters from 3513 reflections
*b* = 6.0426 (5) Å	θ = 2.9–26.0°
*c* = 21.026 (2) Å	µ = 2.41 mm^−^^1^
β = 110.555 (3)°	*T* = 296 K
*V* = 1799.4 (3) Å^3^	Needle, colourless
*Z* = 4	0.20 × 0.15 × 0.10 mm

### (RS)-3-(4-Bromophenyl)-5-[4-(prop-2-ynyloxy)phenyl]-4,5-dihydropyrazole-1-carbothioamide (V) Data collection

**Table:**

Bruker APEXII diffractometer	3365 independent reflections
Radiation source: fine focussealed tube	2559 reflections with *I* > 2σ(*I*)
Graphite monochromator	*R*_int_ = 0.053
φ and ω scans	θ_max_ = 25.5°, θ_min_ = 2.9°
Absorption correction: multi-scan (SADABS; Bruker, 2012)	*h* = −18→18
*T*_min_ = 0.584, *T*_max_ = 0.786	*k* = −7→7
18295 measured reflections	*l* = −25→25

### (RS)-3-(4-Bromophenyl)-5-[4-(prop-2-ynyloxy)phenyl]-4,5-dihydropyrazole-1-carbothioamide (V) Refinement

**Table:**

Refinement on *F*^2^	Primary atom site location: difference Fourier map
Least-squares matrix: full	Hydrogen site location: mixed
*R*[*F*^2^ > 2σ(*F*^2^)] = 0.055	H atoms treated by a mixture of independent and constrained refinement
*wR*(*F*^2^) = 0.113	*w* = 1/[σ^2^(*F*_o_^2^) + (0.026*P*)^2^ + 3.0352*P*] where *P* = (*F*_o_^2^ + 2*F*_c_^2^)/3
*S* = 1.16	(Δ/σ)_max_ < 0.001
3365 reflections	Δρ_max_ = 0.50 e Å^−^^3^
232 parameters	Δρ_min_ = −0.46 e Å^−^^3^
0 restraints	

### (RS)-3-(4-Bromophenyl)-5-[4-(prop-2-ynyloxy)phenyl]-4,5-dihydropyrazole-1-carbothioamide (V) Special details

**Table:**

Geometry. All esds (except the esd in the dihedral angle between two l.s. planes) are estimated using the full covariance matrix. The cell esds are taken into account individually in the estimation of esds in distances, angles and torsion angles; correlations between esds in cell parameters are only used when they are defined by crystal symmetry. An approximate (isotropic) treatment of cell esds is used for estimating esds involving l.s. planes.

### (RS)-3-(4-Bromophenyl)-5-[4-(prop-2-ynyloxy)phenyl]-4,5-dihydropyrazole-1-carbothioamide (V) Fractional atomic coordinates and isotropic or equivalent isotropic displacement parameters (Å^2^)

**Table:**

	*x*	*y*	*z*	*U*_iso_*/*U*_eq_	
N1	0.5170 (2)	0.7430 (5)	0.34130 (16)	0.0407 (8)	
N2	0.4573 (2)	0.5650 (5)	0.31602 (17)	0.0402 (8)	
C3	0.4559 (3)	0.5230 (7)	0.2555 (2)	0.0398 (9)	
C4	0.5136 (3)	0.6835 (7)	0.2315 (2)	0.0451 (10)	
H4A	0.4737	0.7891	0.1994	0.054*	
H4	0.5531	0.6073	0.2108	0.054*	
C5	0.5736 (3)	0.7972 (7)	0.2985 (2)	0.0406 (9)	
H5	0.5759	0.9575	0.2922	0.049*	
C11	0.5228 (3)	0.8343 (6)	0.4015 (2)	0.0387 (9)	
S11	0.59333 (9)	1.05265 (18)	0.43388 (6)	0.0490 (3)	
N11	0.4689 (3)	0.7426 (8)	0.4319 (2)	0.0596 (12)	
H11B	0.467 (4)	0.803 (8)	0.467 (3)	0.071*	
H11A	0.438 (4)	0.632 (9)	0.415 (3)	0.071*	
C31	0.3993 (3)	0.3401 (7)	0.2168 (2)	0.0408 (10)	
C32	0.3746 (4)	0.3310 (8)	0.1462 (2)	0.0583 (13)	
H32	0.3983	0.4365	0.1243	0.070*	
C33	0.3154 (4)	0.1675 (9)	0.1088 (2)	0.0634 (14)	
H33	0.2992	0.1628	0.0619	0.076*	
C34	0.2812 (3)	0.0132 (8)	0.1412 (2)	0.0481 (11)	
Br34	0.19753 (4)	−0.20866 (10)	0.08901 (3)	0.0711 (2)	
C35	0.3073 (3)	0.0108 (8)	0.2113 (2)	0.0522 (11)	
H35	0.2854	−0.0992	0.2330	0.063*	
C36	0.3664 (3)	0.1757 (7)	0.2483 (2)	0.0469 (11)	
H36	0.3845	0.1758	0.2953	0.056*	
C51	0.6719 (3)	0.7015 (7)	0.3267 (2)	0.0392 (9)	
C52	0.6889 (3)	0.5033 (7)	0.3620 (2)	0.0469 (11)	
H52	0.6393	0.4331	0.3703	0.056*	
C53	0.7771 (3)	0.4068 (7)	0.3853 (2)	0.0478 (11)	
H53	0.7865	0.2730	0.4086	0.057*	
C54	0.8517 (3)	0.5110 (7)	0.3737 (2)	0.0414 (10)	
C55	0.8359 (3)	0.7055 (7)	0.3372 (2)	0.0443 (10)	
H55	0.8855	0.7741	0.3284	0.053*	
C56	0.7474 (3)	0.7991 (7)	0.3137 (2)	0.0425 (10)	
H56	0.7378	0.9296	0.2887	0.051*	
O54	0.9434 (2)	0.4368 (5)	0.39764 (15)	0.0508 (8)	
C57	0.9628 (3)	0.2555 (7)	0.4439 (2)	0.0517 (11)	
H57A	0.9304	0.1240	0.4207	0.062*	
H57B	0.9402	0.2887	0.4807	0.062*	
C58	1.0635 (3)	0.2166 (8)	0.4707 (2)	0.0501 (11)	
C59	1.1443 (4)	0.1780 (8)	0.4958 (2)	0.0589 (13)	
H59	1.2084	0.1474	0.5157	0.071*	

### (RS)-3-(4-Bromophenyl)-5-[4-(prop-2-ynyloxy)phenyl]-4,5-dihydropyrazole-1-carbothioamide (V) Atomic displacement parameters (Å^2^)

**Table:**

	*U*^11^	*U*^22^	*U*^33^	*U*^12^	*U*^13^	*U*^23^
N1	0.0450 (19)	0.045 (2)	0.0376 (19)	−0.0074 (16)	0.0215 (16)	−0.0019 (16)
N2	0.0406 (19)	0.0416 (19)	0.039 (2)	−0.0028 (16)	0.0142 (16)	−0.0026 (16)
C3	0.039 (2)	0.044 (2)	0.035 (2)	0.0038 (19)	0.0108 (19)	0.0046 (19)
C4	0.049 (2)	0.052 (3)	0.037 (2)	0.004 (2)	0.019 (2)	0.007 (2)
C5	0.047 (2)	0.039 (2)	0.042 (2)	−0.005 (2)	0.024 (2)	0.0069 (19)
C11	0.040 (2)	0.037 (2)	0.040 (2)	−0.0015 (18)	0.0150 (19)	0.0032 (18)
S11	0.0598 (7)	0.0428 (6)	0.0489 (7)	−0.0136 (5)	0.0247 (6)	−0.0035 (5)
N11	0.071 (3)	0.068 (3)	0.053 (3)	−0.033 (2)	0.038 (2)	−0.019 (2)
C31	0.037 (2)	0.046 (2)	0.038 (2)	0.0051 (19)	0.0122 (19)	−0.0017 (19)
C32	0.067 (3)	0.067 (3)	0.040 (3)	−0.008 (3)	0.018 (2)	0.002 (2)
C33	0.068 (3)	0.078 (4)	0.038 (3)	0.000 (3)	0.011 (3)	−0.007 (3)
C34	0.036 (2)	0.051 (3)	0.054 (3)	0.000 (2)	0.012 (2)	−0.017 (2)
Br34	0.0495 (3)	0.0782 (4)	0.0824 (4)	−0.0050 (3)	0.0191 (3)	−0.0385 (3)
C35	0.052 (3)	0.050 (3)	0.059 (3)	−0.003 (2)	0.024 (2)	−0.006 (2)
C36	0.047 (2)	0.052 (3)	0.043 (3)	0.001 (2)	0.017 (2)	−0.002 (2)
C51	0.047 (2)	0.037 (2)	0.038 (2)	−0.0083 (19)	0.019 (2)	−0.0013 (18)
C52	0.046 (3)	0.044 (2)	0.058 (3)	−0.008 (2)	0.027 (2)	0.010 (2)
C53	0.053 (3)	0.039 (2)	0.055 (3)	−0.002 (2)	0.022 (2)	0.010 (2)
C54	0.045 (2)	0.045 (2)	0.038 (2)	−0.006 (2)	0.019 (2)	−0.0059 (19)
C55	0.046 (2)	0.051 (3)	0.042 (2)	−0.011 (2)	0.023 (2)	0.000 (2)
C56	0.054 (3)	0.041 (2)	0.036 (2)	−0.010 (2)	0.021 (2)	0.0056 (19)
O54	0.0446 (17)	0.061 (2)	0.0516 (18)	0.0007 (15)	0.0223 (15)	0.0057 (15)
C57	0.053 (3)	0.049 (3)	0.055 (3)	−0.002 (2)	0.021 (2)	−0.003 (2)
C58	0.057 (3)	0.055 (3)	0.042 (3)	−0.002 (2)	0.021 (2)	−0.009 (2)
C59	0.055 (3)	0.072 (3)	0.052 (3)	0.005 (3)	0.023 (3)	0.003 (3)

### (RS)-3-(4-Bromophenyl)-5-[4-(prop-2-ynyloxy)phenyl]-4,5-dihydropyrazole-1-carbothioamide (V) Geometric parameters (Å, º)

**Table:**

N1—C11	1.354 (5)	C34—Br34	1.904 (4)
N1—N2	1.385 (4)	C35—C36	1.381 (6)
N1—C5	1.480 (5)	C35—H35	0.9300
N2—C3	1.292 (5)	C36—H36	0.9300
C3—C31	1.458 (6)	C51—C52	1.385 (6)
C3—C4	1.505 (5)	C51—C56	1.396 (5)
C4—C5	1.542 (6)	C52—C53	1.378 (6)
C4—H4A	0.9700	C52—H52	0.9300
C4—H4	0.9700	C53—C54	1.387 (6)
C5—C51	1.509 (6)	C53—H53	0.9300
C5—H5	0.9800	C54—O54	1.374 (5)
C11—N11	1.323 (5)	C54—C55	1.378 (6)
C11—S11	1.683 (4)	C55—C56	1.375 (6)
N11—H11B	0.83 (5)	C55—H55	0.9300
N11—H11A	0.82 (5)	C56—H56	0.9300
C31—C36	1.380 (6)	O54—C57	1.425 (5)
C31—C32	1.398 (6)	C57—C58	1.446 (6)
C32—C33	1.379 (7)	C57—H57A	0.9700
C32—H32	0.9300	C57—H57B	0.9700
C33—C34	1.360 (6)	C58—C59	1.172 (6)
C33—H33	0.9300	C59—H59	0.9300
C34—C35	1.387 (6)		
			
C11—N1—N2	119.6 (3)	C33—C34—Br34	119.2 (4)
C11—N1—C5	128.0 (3)	C35—C34—Br34	119.3 (4)
N2—N1—C5	112.1 (3)	C36—C35—C34	118.5 (4)
C3—N2—N1	108.2 (3)	C36—C35—H35	120.8
N2—C3—C31	120.1 (4)	C34—C35—H35	120.8
N2—C3—C4	113.1 (4)	C31—C36—C35	121.4 (4)
C31—C3—C4	126.7 (4)	C31—C36—H36	119.3
C3—C4—C5	101.9 (3)	C35—C36—H36	119.3
C3—C4—H4A	111.4	C52—C51—C56	117.3 (4)
C5—C4—H4A	111.4	C52—C51—C5	121.0 (3)
C3—C4—H4	111.4	C56—C51—C5	121.5 (4)
C5—C4—H4	111.4	C53—C52—C51	122.0 (4)
H4A—C4—H4	109.3	C53—C52—H52	119.0
N1—C5—C51	112.1 (3)	C51—C52—H52	119.0
N1—C5—C4	100.2 (3)	C52—C53—C54	119.5 (4)
C51—C5—C4	111.7 (3)	C52—C53—H53	120.3
N1—C5—H5	110.8	C54—C53—H53	120.3
C51—C5—H5	110.8	O54—C54—C55	116.0 (3)
C4—C5—H5	110.8	O54—C54—C53	124.5 (4)
N11—C11—N1	115.6 (4)	C55—C54—C53	119.5 (4)
N11—C11—S11	122.9 (3)	C56—C55—C54	120.5 (4)
N1—C11—S11	121.5 (3)	C56—C55—H55	119.7
C11—N11—H11B	117 (4)	C54—C55—H55	119.7
C11—N11—H11A	119 (4)	C55—C56—C51	121.1 (4)
H11B—N11—H11A	124 (5)	C55—C56—H56	119.5
C36—C31—C32	118.2 (4)	C51—C56—H56	119.5
C36—C31—C3	121.2 (4)	C54—O54—C57	116.1 (3)
C32—C31—C3	120.5 (4)	O54—C57—C58	109.1 (4)
C33—C32—C31	120.9 (5)	O54—C57—H57A	109.9
C33—C32—H32	119.6	C58—C57—H57A	109.9
C31—C32—H32	119.6	O54—C57—H57B	109.9
C34—C33—C32	119.4 (4)	C58—C57—H57B	109.9
C34—C33—H33	120.3	H57A—C57—H57B	108.3
C32—C33—H33	120.3	C59—C58—C57	175.8 (5)
C33—C34—C35	121.5 (4)	C58—C59—H59	180.0
			
C11—N1—N2—C3	173.1 (4)	C32—C33—C34—Br34	178.7 (4)
C5—N1—N2—C3	−11.5 (4)	C33—C34—C35—C36	2.7 (7)
N1—N2—C3—C31	179.2 (3)	Br34—C34—C35—C36	−178.7 (3)
N1—N2—C3—C4	−3.1 (5)	C32—C31—C36—C35	−2.7 (6)
N2—C3—C4—C5	15.1 (5)	C3—C31—C36—C35	175.3 (4)
C31—C3—C4—C5	−167.3 (4)	C34—C35—C36—C31	0.1 (6)
C11—N1—C5—C51	76.1 (5)	N1—C5—C51—C52	32.2 (5)
N2—N1—C5—C51	−98.8 (4)	C4—C5—C51—C52	−79.4 (5)
C11—N1—C5—C4	−165.2 (4)	N1—C5—C51—C56	−153.0 (4)
N2—N1—C5—C4	19.8 (4)	C4—C5—C51—C56	95.5 (4)
C3—C4—C5—N1	−19.2 (4)	C56—C51—C52—C53	1.6 (6)
C3—C4—C5—C51	99.6 (4)	C5—C51—C52—C53	176.6 (4)
N2—N1—C11—N11	−0.1 (6)	C51—C52—C53—C54	0.5 (7)
C5—N1—C11—N11	−174.8 (4)	C52—C53—C54—O54	176.2 (4)
N2—N1—C11—S11	−179.2 (3)	C52—C53—C54—C55	−2.1 (6)
C5—N1—C11—S11	6.1 (6)	O54—C54—C55—C56	−177.0 (4)
N2—C3—C31—C36	−17.0 (6)	C53—C54—C55—C56	1.5 (6)
C4—C3—C31—C36	165.6 (4)	C54—C55—C56—C51	0.7 (6)
N2—C3—C31—C32	161.0 (4)	C52—C51—C56—C55	−2.2 (6)
C4—C3—C31—C32	−16.4 (6)	C5—C51—C56—C55	−177.2 (4)
C36—C31—C32—C33	2.8 (7)	C55—C54—O54—C57	170.7 (4)
C3—C31—C32—C33	−175.3 (4)	C53—C54—O54—C57	−7.6 (6)
C31—C32—C33—C34	−0.2 (8)	C54—O54—C57—C58	−172.7 (3)
C32—C33—C34—C35	−2.6 (7)		

### (RS)-3-(4-Bromophenyl)-5-[4-(prop-2-ynyloxy)phenyl]-4,5-dihydropyrazole-1-carbothioamide (V) Hydrogen-bond geometry (Å, º)

**Table:**

*D*—H···*A*	*D*—H	H···*A*	*D*···*A*	*D*—H···*A*
N11—H11*A*···N2	0.82 (5)	2.24 (6)	2.611 (5)	108 (5)
N11—H11*A*···Br34^i^	0.82 (5)	2.89 (6)	3.632 (5)	152 (5)
N11—H11*B*···S11^ii^	0.83 (6)	2.70 (6)	3.500 (5)	162 (6)
C52—H52···S11^iii^	0.93	2.87	3.650 (5)	143

Symmetry codes: (i) −*x*+1/2, *y*+1/2, −*z*+1/2; (ii) −*x*+1, −*y*+2, −*z*+1; (iii) *x*, *y*−1, *z*.

### (RS)-3-(4-Methoxyphenyl)-5-[4-(prop-2-ynyloxy)phenyl]-4,5-dihydropyrazole-1-carbothioamide (VI) Crystal data

**Table:**

C_20_H_19_N_3_O_2_S	*F*(000) = 768
*M**_r_* = 365.44	*D*_x_ = 1.330 Mg m^−^^3^
Monoclinic, *P*2_1_/*c*	Mo *K*α radiation, λ = 0.71073 Å
*a* = 11.7852 (15) Å	Cell parameters from 3824 reflections
*b* = 7.5345 (11) Å	θ = 1.7–26.6°
*c* = 20.599 (3) Å	µ = 0.20 mm^−^^1^
β = 93.555 (4)°	*T* = 296 K
*V* = 1825.6 (4) Å^3^	Block, colourless
*Z* = 4	0.20 × 0.20 × 0.15 mm

### (RS)-3-(4-Methoxyphenyl)-5-[4-(prop-2-ynyloxy)phenyl]-4,5-dihydropyrazole-1-carbothioamide (VI) Data collection

**Table:**

Bruker APEXII diffractometer	3822 independent reflections
Radiation source: fine focussealed tube	1864 reflections with *I* > 2σ(*I*)
Graphite monochromator	*R*_int_ = 0.100
φ and ω scans	θ_max_ = 26.6°, θ_min_ = 2.6°
Absorption correction: multi-scan (SADABS; Bruker, 2012)	*h* = −14→14
*T*_min_ = 0.908, *T*_max_ = 0.971	*k* = −9→9
20467 measured reflections	*l* = −23→25

### (RS)-3-(4-Methoxyphenyl)-5-[4-(prop-2-ynyloxy)phenyl]-4,5-dihydropyrazole-1-carbothioamide (VI) Refinement

**Table:**

Refinement on *F*^2^	Primary atom site location: difference Fourier map
Least-squares matrix: full	Hydrogen site location: mixed
*R*[*F*^2^ > 2σ(*F*^2^)] = 0.051	H atoms treated by a mixture of independent and constrained refinement
*wR*(*F*^2^) = 0.118	*w* = 1/[σ^2^(*F*_o_^2^) + (0.0424*P*)^2^] where *P* = (*F*_o_^2^ + 2*F*_c_^2^)/3
*S* = 0.97	(Δ/σ)_max_ < 0.001
3822 reflections	Δρ_max_ = 0.21 e Å^−^^3^
242 parameters	Δρ_min_ = −0.24 e Å^−^^3^
0 restraints	

### (RS)-3-(4-Methoxyphenyl)-5-[4-(prop-2-ynyloxy)phenyl]-4,5-dihydropyrazole-1-carbothioamide (VI) Special details

**Table:**

Geometry. All esds (except the esd in the dihedral angle between two l.s. planes) are estimated using the full covariance matrix. The cell esds are taken into account individually in the estimation of esds in distances, angles and torsion angles; correlations between esds in cell parameters are only used when they are defined by crystal symmetry. An approximate (isotropic) treatment of cell esds is used for estimating esds involving l.s. planes.

### (RS)-3-(4-Methoxyphenyl)-5-[4-(prop-2-ynyloxy)phenyl]-4,5-dihydropyrazole-1-carbothioamide (VI) Fractional atomic coordinates and isotropic or equivalent isotropic displacement parameters (Å^2^)

**Table:**

	*x*	*y*	*z*	*U*_iso_*/*U*_eq_	
N1	0.32812 (16)	0.5906 (3)	0.30356 (11)	0.0359 (6)	
N2	0.39265 (16)	0.4472 (3)	0.32813 (11)	0.0359 (6)	
C3	0.3292 (2)	0.3478 (3)	0.36206 (13)	0.0358 (7)	
C4	0.2109 (2)	0.4218 (4)	0.36538 (14)	0.0431 (8)	
H4A	0.2006	0.4759	0.4073	0.052*	
H4B	0.1538	0.3303	0.3573	0.052*	
C5	0.20546 (19)	0.5611 (3)	0.31084 (15)	0.0396 (7)	
H5	0.1699	0.6702	0.3257	0.047*	
C11	0.3763 (2)	0.7190 (3)	0.26933 (13)	0.0341 (7)	
S11	0.29954 (5)	0.88567 (9)	0.23445 (4)	0.0462 (3)	
N11	0.48918 (18)	0.7072 (3)	0.26592 (12)	0.0398 (7)	
H11A	0.529 (2)	0.614 (3)	0.2788 (13)	0.048*	
H11B	0.520 (2)	0.781 (3)	0.2386 (13)	0.048*	
C31	0.3722 (2)	0.1830 (3)	0.39094 (13)	0.0375 (7)	
C32	0.3004 (2)	0.0594 (4)	0.41761 (14)	0.0452 (8)	
H32	0.2243	0.0881	0.4213	0.054*	
C33	0.3393 (2)	−0.1033 (4)	0.43855 (14)	0.0499 (8)	
H33	0.2898	−0.1834	0.4563	0.060*	
C34	0.4514 (3)	−0.1485 (4)	0.43337 (14)	0.0456 (8)	
C35	0.5260 (2)	−0.0259 (4)	0.40995 (14)	0.0444 (8)	
H35	0.6026	−0.0541	0.4083	0.053*	
C36	0.4869 (2)	0.1379 (3)	0.38909 (14)	0.0414 (7)	
H36	0.5376	0.2198	0.3735	0.050*	
O34	0.48126 (17)	−0.3164 (3)	0.45311 (10)	0.0614 (6)	
C37	0.5942 (3)	−0.3754 (4)	0.44406 (16)	0.0660 (10)	
H37A	0.6085	−0.3691	0.3987	0.099*	
H37B	0.6028	−0.4958	0.4588	0.099*	
H37C	0.6474	−0.3010	0.4685	0.099*	
C51	0.1451 (2)	0.4974 (3)	0.24816 (14)	0.0350 (7)	
C52	0.1993 (2)	0.4027 (4)	0.20147 (15)	0.0476 (8)	
H52	0.2764	0.3777	0.2083	0.057*	
C53	0.1417 (2)	0.3442 (4)	0.14482 (15)	0.0493 (8)	
H53	0.1798	0.2815	0.1140	0.059*	
C54	0.0272 (2)	0.3798 (3)	0.13462 (15)	0.0394 (7)	
C55	−0.0289 (2)	0.4687 (3)	0.18155 (15)	0.0408 (8)	
H55	−0.1065	0.4899	0.1754	0.049*	
C56	0.0297 (2)	0.5260 (3)	0.23739 (15)	0.0406 (8)	
H56	−0.0094	0.5855	0.2687	0.049*	
O54	−0.03798 (14)	0.3313 (3)	0.07948 (10)	0.0550 (6)	
C57	0.0190 (3)	0.2607 (4)	0.02690 (16)	0.0612 (9)	
H57A	0.0538	0.1482	0.0395	0.073*	
H57B	0.0787	0.3414	0.0154	0.073*	
C58	−0.0612 (3)	0.2348 (4)	−0.02888 (19)	0.0612 (10)	
C59	−0.1219 (3)	0.2176 (5)	−0.0749 (2)	0.0854 (12)	
H59	−0.1707	0.2038	−0.1119	0.102*	

### (RS)-3-(4-Methoxyphenyl)-5-[4-(prop-2-ynyloxy)phenyl]-4,5-dihydropyrazole-1-carbothioamide (VI) Atomic displacement parameters (Å^2^)

**Table:**

	*U*^11^	*U*^22^	*U*^33^	*U*^12^	*U*^13^	*U*^23^
N1	0.0287 (12)	0.0365 (13)	0.0423 (17)	−0.0015 (10)	0.0015 (11)	0.0019 (12)
N2	0.0345 (12)	0.0368 (13)	0.0361 (16)	−0.0009 (11)	−0.0003 (11)	0.0000 (11)
C3	0.0376 (15)	0.0399 (17)	0.0298 (19)	−0.0063 (13)	0.0020 (13)	−0.0049 (14)
C4	0.0382 (16)	0.0501 (18)	0.042 (2)	−0.0060 (13)	0.0076 (14)	−0.0019 (16)
C5	0.0267 (14)	0.0425 (17)	0.050 (2)	−0.0007 (13)	0.0072 (14)	−0.0051 (16)
C11	0.0336 (15)	0.0344 (15)	0.0344 (19)	−0.0045 (13)	0.0017 (13)	−0.0058 (14)
S11	0.0388 (4)	0.0429 (4)	0.0565 (6)	0.0027 (3)	0.0007 (4)	0.0048 (4)
N11	0.0334 (14)	0.0378 (15)	0.0482 (19)	0.0000 (11)	0.0028 (12)	0.0077 (13)
C31	0.0434 (16)	0.0404 (17)	0.0287 (19)	−0.0072 (14)	0.0021 (13)	0.0002 (14)
C32	0.0457 (16)	0.056 (2)	0.034 (2)	−0.0095 (15)	0.0025 (15)	0.0001 (16)
C33	0.062 (2)	0.0484 (19)	0.039 (2)	−0.0154 (16)	0.0031 (16)	0.0061 (16)
C34	0.061 (2)	0.0429 (19)	0.032 (2)	−0.0076 (16)	−0.0019 (15)	0.0014 (15)
C35	0.0454 (17)	0.0462 (19)	0.041 (2)	−0.0009 (15)	−0.0015 (15)	0.0031 (16)
C36	0.0452 (17)	0.0422 (18)	0.037 (2)	−0.0067 (14)	0.0016 (14)	0.0024 (15)
O34	0.0796 (15)	0.0455 (13)	0.0592 (17)	−0.0021 (11)	0.0061 (12)	0.0134 (12)
C37	0.087 (2)	0.048 (2)	0.063 (3)	0.0078 (18)	0.000 (2)	0.0058 (18)
C51	0.0295 (14)	0.0343 (15)	0.041 (2)	−0.0005 (12)	0.0029 (14)	0.0024 (14)
C52	0.0303 (15)	0.0586 (19)	0.054 (2)	0.0087 (14)	0.0045 (15)	−0.0084 (18)
C53	0.0416 (17)	0.063 (2)	0.044 (2)	0.0055 (15)	0.0041 (15)	−0.0163 (17)
C54	0.0345 (16)	0.0405 (17)	0.043 (2)	−0.0038 (13)	−0.0021 (15)	0.0003 (15)
C55	0.0283 (14)	0.0414 (17)	0.052 (2)	0.0005 (13)	0.0005 (15)	−0.0002 (16)
C56	0.0325 (15)	0.0390 (16)	0.051 (2)	0.0005 (13)	0.0083 (15)	−0.0066 (15)
O54	0.0442 (12)	0.0752 (15)	0.0446 (16)	−0.0039 (10)	−0.0042 (11)	−0.0093 (12)
C57	0.067 (2)	0.063 (2)	0.053 (3)	0.0021 (18)	−0.0025 (19)	−0.0076 (19)
C58	0.075 (2)	0.054 (2)	0.053 (3)	0.0026 (18)	−0.009 (2)	−0.0034 (19)
C59	0.111 (3)	0.075 (3)	0.066 (3)	0.001 (2)	−0.029 (2)	−0.005 (2)

### (RS)-3-(4-Methoxyphenyl)-5-[4-(prop-2-ynyloxy)phenyl]-4,5-dihydropyrazole-1-carbothioamide (VI) Geometric parameters (Å, º)

**Table:**

N1—C11	1.343 (3)	C35—H35	0.9300
N1—N2	1.398 (3)	C36—H36	0.9300
N1—C5	1.479 (3)	O34—C37	1.426 (3)
N2—C3	1.294 (3)	C37—H37A	0.9600
C3—C31	1.455 (3)	C37—H37B	0.9600
C3—C4	1.507 (3)	C37—H37C	0.9600
C4—C5	1.536 (4)	C51—C56	1.381 (3)
C4—H4A	0.9700	C51—C52	1.385 (3)
C4—H4B	0.9700	C52—C53	1.385 (4)
C5—C51	1.513 (4)	C52—H52	0.9300
C5—H5	0.9800	C53—C54	1.379 (3)
C11—N11	1.339 (3)	C53—H53	0.9300
C11—S11	1.683 (3)	C54—C55	1.378 (4)
N11—H11A	0.88 (2)	C54—O54	1.380 (3)
N11—H11B	0.89 (3)	C55—C56	1.374 (4)
C31—C32	1.394 (3)	C55—H55	0.9300
C31—C36	1.396 (3)	C56—H56	0.9300
C32—C33	1.369 (4)	O54—C57	1.413 (3)
C32—H32	0.9300	C57—C58	1.455 (4)
C33—C34	1.375 (4)	C57—H57A	0.9700
C33—H33	0.9300	C57—H57B	0.9700
C34—O34	1.368 (3)	C58—C59	1.160 (4)
C34—C35	1.383 (3)	C59—H59	0.9300
C35—C36	1.377 (3)		
			
C11—N1—N2	120.55 (19)	C34—C35—H35	120.0
C11—N1—C5	127.5 (2)	C35—C36—C31	121.0 (2)
N2—N1—C5	111.11 (19)	C35—C36—H36	119.5
C3—N2—N1	108.86 (19)	C31—C36—H36	119.5
N2—C3—C31	121.1 (2)	C34—O34—C37	118.3 (2)
N2—C3—C4	112.2 (2)	O34—C37—H37A	109.5
C31—C3—C4	126.7 (2)	O34—C37—H37B	109.5
C3—C4—C5	102.5 (2)	H37A—C37—H37B	109.5
C3—C4—H4A	111.3	O34—C37—H37C	109.5
C5—C4—H4A	111.3	H37A—C37—H37C	109.5
C3—C4—H4B	111.3	H37B—C37—H37C	109.5
C5—C4—H4B	111.3	C56—C51—C52	117.4 (3)
H4A—C4—H4B	109.2	C56—C51—C5	119.6 (2)
N1—C5—C51	111.8 (2)	C52—C51—C5	122.9 (2)
N1—C5—C4	100.4 (2)	C51—C52—C53	121.7 (2)
C51—C5—C4	113.8 (2)	C51—C52—H52	119.1
N1—C5—H5	110.2	C53—C52—H52	119.1
C51—C5—H5	110.2	C54—C53—C52	119.3 (3)
C4—C5—H5	110.2	C54—C53—H53	120.3
N11—C11—N1	115.7 (2)	C52—C53—H53	120.3
N11—C11—S11	122.4 (2)	C55—C54—C53	119.6 (3)
N1—C11—S11	121.85 (18)	C55—C54—O54	116.1 (2)
C11—N11—H11A	123.2 (16)	C53—C54—O54	124.3 (3)
C11—N11—H11B	116.0 (16)	C56—C55—C54	120.2 (2)
H11A—N11—H11B	118 (2)	C56—C55—H55	119.9
C32—C31—C36	117.4 (3)	C54—C55—H55	119.9
C32—C31—C3	121.7 (2)	C55—C56—C51	121.6 (3)
C36—C31—C3	120.7 (2)	C55—C56—H56	119.2
C33—C32—C31	121.5 (3)	C51—C56—H56	119.2
C33—C32—H32	119.3	C54—O54—C57	117.6 (2)
C31—C32—H32	119.3	O54—C57—C58	109.9 (3)
C32—C33—C34	120.1 (3)	O54—C57—H57A	109.7
C32—C33—H33	119.9	C58—C57—H57A	109.7
C34—C33—H33	119.9	O54—C57—H57B	109.7
O34—C34—C33	115.9 (3)	C58—C57—H57B	109.7
O34—C34—C35	124.3 (3)	H57A—C57—H57B	108.2
C33—C34—C35	119.8 (3)	C59—C58—C57	177.2 (4)
C36—C35—C34	120.0 (3)	C58—C59—H59	180.0
C36—C35—H35	120.0		
			
C11—N1—N2—C3	176.5 (2)	O34—C34—C35—C36	177.7 (3)
C5—N1—N2—C3	−13.2 (3)	C33—C34—C35—C36	−3.0 (4)
N1—N2—C3—C31	176.8 (2)	C34—C35—C36—C31	−0.2 (4)
N1—N2—C3—C4	−1.8 (3)	C32—C31—C36—C35	3.1 (4)
N2—C3—C4—C5	15.0 (3)	C3—C31—C36—C35	−172.9 (3)
C31—C3—C4—C5	−163.6 (3)	C33—C34—O34—C37	175.1 (3)
C11—N1—C5—C51	69.8 (3)	C35—C34—O34—C37	−5.5 (4)
N2—N1—C5—C51	−99.7 (2)	N1—C5—C51—C56	−155.6 (2)
C11—N1—C5—C4	−169.2 (2)	C4—C5—C51—C56	91.5 (3)
N2—N1—C5—C4	21.3 (3)	N1—C5—C51—C52	27.6 (4)
C3—C4—C5—N1	−20.3 (3)	C4—C5—C51—C52	−85.3 (3)
C3—C4—C5—C51	99.3 (2)	C56—C51—C52—C53	2.4 (4)
N2—N1—C11—N11	−5.7 (4)	C5—C51—C52—C53	179.2 (3)
C5—N1—C11—N11	−174.3 (2)	C51—C52—C53—C54	−0.5 (5)
N2—N1—C11—S11	175.50 (18)	C52—C53—C54—C55	−1.7 (4)
C5—N1—C11—S11	6.9 (4)	C52—C53—C54—O54	178.6 (3)
N2—C3—C31—C32	−168.5 (3)	C53—C54—C55—C56	1.9 (4)
C4—C3—C31—C32	10.0 (4)	O54—C54—C55—C56	−178.4 (2)
N2—C3—C31—C36	7.4 (4)	C54—C55—C56—C51	0.2 (4)
C4—C3—C31—C36	−174.2 (3)	C52—C51—C56—C55	−2.3 (4)
C36—C31—C32—C33	−2.9 (4)	C5—C51—C56—C55	−179.2 (2)
C3—C31—C32—C33	173.1 (3)	C55—C54—O54—C57	172.0 (2)
C31—C32—C33—C34	−0.2 (5)	C53—C54—O54—C57	−8.3 (4)
C32—C33—C34—O34	−177.4 (3)	C54—O54—C57—C58	−174.1 (2)
C32—C33—C34—C35	3.2 (4)		

### (RS)-3-(4-Methoxyphenyl)-5-[4-(prop-2-ynyloxy)phenyl]-4,5-dihydropyrazole-1-carbothioamide (VI) Hydrogen-bond geometry (Å, º)

**Table:**

*D*—H···*A*	*D*—H	H···*A*	*D*···*A*	*D*—H···*A*
N11—H11*A*···N2	0.88 (2)	2.32 (2)	2.637 (3)	101.2 (18)
N11—H11*A*···S11^i^	0.88 (2)	2.68 (2)	3.474 (2)	151 (2)
N11—H11*B*···N2^ii^	0.89 (2)	2.17 (2)	3.049 (3)	175 (2)
C37—H37*B*···O34^iii^	0.96	2.55	3.302 (4)	135
C56—H56···*Cg*2^iv^	0.93	2.93	3.717 (3)	143

Symmetry codes: (i) −*x*+1, *y*−1/2, −*z*+1/2; (ii) −*x*+1, *y*+1/2, −*z*+1/2; (iii) −*x*+1, −*y*−1, −*z*+1; (iv) −*x*, *y*+1/2, −*z*+1/2.
